# Genome-Based Taxonomic Rearrangement of the Order *Geobacterales* Including the Description of *Geomonas azotofigens* sp. nov. and *Geomonas diazotrophica* sp. nov.

**DOI:** 10.3389/fmicb.2021.737531

**Published:** 2021-09-30

**Authors:** Zhenxing Xu, Yoko Masuda, Xueding Wang, Natsumi Ushijima, Yutaka Shiratori, Keishi Senoo, Hideomi Itoh

**Affiliations:** ^1^Department of Applied Biological Chemistry, Graduate School of Agricultural and Life Sciences, The University of Tokyo, Tokyo, Japan; ^2^Support Section for Education and Research, Graduate School of Dental Medicine, Hokkaido University, Hokkaido, Japan; ^3^Niigata Agricultural Research Institute, Niigata, Japan; ^4^Collaborative Research Institute for Innovative Microbiology, The University of Tokyo, Tokyo, Japan; ^5^Bioproduction Research Institute, National Institute of Advanced Industrial Science and Technology (AIST) Hokkaido, Hokkaido, Japan

**Keywords:** *Geobacterales*, taxonomic reassignment, genome, *Geomonas azotofigens*, *Geomonas diazotrophica*

## Abstract

*Geobacterales* is a recently proposed order comprising members who originally belonged to the well-known family *Geobacteraceae*, which is a key group in terrestrial ecosystems involved in biogeochemical cycles and has been widely investigated in bioelectrochemistry and bioenergy fields. Previous studies have illustrated the taxonomic structure of most members in this group based on genomic phylogeny; however, several members are still in a pendent or chaotic taxonomic status owing to the lack of genome sequences. To address this issue, we performed this taxonomic reassignment using currently available genome sequences, along with the description of two novel paddy soil-isolated strains, designated Red51^T^ and Red69^T^, which are phylogenetically located within this order. Phylogenomic analysis based on 120 ubiquitous single-copy proteins robustly separated the species *Geobacter luticola* from other known genera and placed the genus *Oryzomonas* (fam. *Geobacteraceae*) into the family ‘*Pseudopelobacteraceae*’; thus, a novel genus *Geomobilimonas* is proposed, and the family ‘*Pseudopelobacteraceae*’ was emended. Moreover, genomic comparisons with similarity indexes, including average amino acid identity (AAI), percentage of conserved protein (POCP), and average nucleotide identity (ANI), showed proper thresholds as genera boundaries in this order with values of 70%, 65%, and 74% for AAI, POCP, and ANI, respectively. Based on this, the three species *Geobacter argillaceus*, *Geobacter pelophilus*, and *Geobacter chapellei* should be three novel genera, for which the names *Geomobilibacter*, *Geoanaerobacter*, and *Pelotalea* are proposed, respectively. In addition, the two novel isolated strains phylogenetically belonged to the genus *Geomonas*, family *Geobacteraceae*, and shared genomic similarity values higher than those of genera boundaries, but lower than those of species boundaries with each other and their neighbors. Taken together with phenotypic and chemotaxonomic characteristics similar to other *Geomonas* species, these two strains, Red51^T^ and Red69^T^, represent two novel species in the genus *Geomonas*, for which the names *Geomonas azotofigens* sp. nov. and *Geomonas diazotrophica* sp. nov. are proposed, respectively.

## Introduction

The order *Geobacterales*, belonging to the class *Desulfuromonadia* of the phylum *Desulfobacterota*, was recently proposed by Waite et al. (2020) based on multigene phylogenomic analysis and includes members that originally belonged to the well-known family *Geobacteraceae*. At the time of writing, this order includes two families: *Geobacteraceae* (genera: *Geobacter*, *Geomonas*, *Geotalea*, and *Oryzomonas*) and ‘*Pseudopelobacteraceae*’ (genera: *Trichlorobacter* and ‘*Pseudopelobacter*’) with *Geobacter* as the type genus. Notably, the taxonomic classification of the family *Geobacteraceae* has a tortuous history, with many of its members being uprooted and renamed multiple times. This family was first proposed by Holmes et al. (2004a) in the class *Deltaproteobacteria* but pendent in the order level at that time, consisting of five genera *Geobacter*, *Desulfuromonas*, *Desulfuromusa*, *Pelobacter*, and *Malonomonas*, based on the phylogeny of six housekeeping genes (16S rRNA gene, *gyrB*, *recA*, *nifD*, *fusA*, and *rpoB*). Subsequently, all of these members were separated into two families, *Geobacteraceae* and *Desulfuromonadaceae*. Garrity et al. (2005) emended the description of *Geobacteraceae* (Valid publication: Euzéby, 2006), containing the genus *Geobacter* and a single species, *Trichlorobacter thiogenes*, which was later renamed as *Geobacter thiogenes*, based on the phylogenetic and physiological comparison to the genus *Geobacter* (Nevin et al., 2007), whereas Kuever et al. (2005) proposed the family *Desulfuromonadaceae* (Valid publication: Euzéby, 2006), containing the other four genera *Desulfuromonas*, *Desulfuromusa*, *Pelobacter,* and *Malonomonas*. Concurrently, these two families were assigned into the order *Desulfuromonadales* of the class *Deltaproteobacteria* (Garrity et al., 2005; Kuever et al., 2005; Euzéby, 2006). In addition, four novel genera *Geothermobacter*, *Geopsychrobacter*, *Geomonas*, and *Oryzomonas* were then proposed and assigned into the family *Geobacteraceae* (Kashefi et al., 2003; Holmes et al., 2004b; Xu et al., 2019, 2020), indicating the five genera, i.e., *Geobacter*, *Geothermobacter*, *Geopsychrobacter*, *Geomonas,* and *Oryzomonas*, comprised the family *Geobacteraceae* at that time. Although the two genera *Geothermobacter* and *Geopsychrobacter* nominally belong to the family *Geobacteraceae*, their phylogenetic and phenotypic characteristics are more closely aligned with the other family, *Desulfuromonadaceae* (Röling, 2014).

Recently, a reclassification study focusing on resolving the taxonomic conflicts of species in the class *Deltaproteobacteria* was performed using the phylogenetic analysis of 120 conserved single-copy marker genes and rRNA genes (Waite et al., 2020). Based on this, the two genera *Geothermobacter* and *Geopsychrobacter* have been reclassified as two novel families *Geothermobacteraceae* and *Geopsychrobacteraceae*, respectively, and were reassigned into the order *Desulfuromonadales*, whereas the species in the genus *Geobacter* together with the species *Pelobacter propionicus* were reclassified into two families *Geobacteraceae* and ‘*Pseudopelobacteraceae*’, and formed a novel order, *Geobacterales*. This reclassification work has restored the taxonomic order of most species in the original family *Geobacteraceae*; however, the new taxon was proposed based on a phylogenomic analysis of multiple marker genes without considering genome similarity, which is also key evidence confirming the taxonomic positions (Chun et al., 2018), causing the taxonomic controversy of several species in this group. Moreover, the taxonomic status of the two species, *Geobacter luticola* and *Geobacter argillaceus*, was not reclassified in that analysis because of the absence of genomic information, resulting in the pendent taxonomic status of these two species. Besides, the two recently proposed genera *Geomonas* and *Oryzomonas* (Xu et al., 2019, 2020), belonging to the original family *Geobacteraceae*, were not considered in Waite’s reclassification work because of the nearly parallel time of description, leading to the confused taxonomic description of all species in these two genera. These facts suggest that further remediation for the taxonomic description of several species in the order *Geobacterales* is clearly required.

As the members of the order *Geobacterales* were originally treated as *Geobacter* species, the common physiological characteristics of this order are Gram-stain negative, rod-shaped, red-pigmented, and obligate anaerobic, as well as extensively participating in biogeochemical processes, such as nitrogen fixation, dissimilatory nitrate reduction to ammonium, Fe(III)-reduction along with acetate utilization, and heavy metal [U(VI), Co(III), and Mn(IV)] reduction in various environments (Lovley et al., 2011; Masuda et al., 2017). Moreover, several members of this order have been investigated widely in bioelectrochemistry and bioenergy fields, especially as the major model microorganisms of microbial fuel cells, owing to their ability to produce conductive pili as nanowires for discharging respiratory electrons to solid-phase electron acceptors and radionuclides, or for wiring cells in current-harvesting biofilms (Shi et al., 2016; Reguera and Kashefi, 2019). These facts indicate the important and intriguing features of the strains in this group and thus call for the target isolation of this bacterial group. *Geomonas* is a recently proposed genus that is separated from the genus *Geobacter* within the family *Geobacteraceae,* and currently comprise nine validly published species^1^ (Xu et al., 2019). Notably, a recently proposed genus, *Citrifermentans*, contained the same species as *Geomonas* (Waite et al., 2020), but it is a later synonym of *Geomonas* (Xu et al., 2020); thus, *Geomonas* is the correct name to address species in the order *Geobacterales* (Sanford et al., 2021). In this study, there are two bacterial strains, designated Red51^T^ and Red69^T^, newly isolated from paddy soils collected from two different fields, which shared a relatively high 16S rRNA gene similarity to the type strains in the genus *Geomonas*. Therefore, we performed this study aiming to re-settle the taxonomic status of species in the order *Geobacterales* and describe two novel species within this order.

## Materials and Methods

### Bacterial Isolation and Culture

Strains Red51^T^ and Red69^T^ were recovered from anaerobic enrichment cultures containing microbe-harboring paddy soils collected from Yukuhashi, Fukuoka, Kanzaki, and Saga, Japan, respectively. The detailed enrichment method by using the soil slurry incubation was the same as that introduced in our previous studies (Xu et al., 2020; Itoh et al., 2021). After isolation and purification processes, the two strains were routinely cultured on R2A agar (Difco, NJ, United States) plates supplemented with 5mM fumarate (modified R2A agar) or R2A broth (Wako, Japan) supplemented with 5mM fumarate (modified R2A broth). In addition, these two strains were also found to grow well in modified freshwater medium (MFM) with 10mM fumarate and 20mM acetate as the electron acceptor and donor, respectively (Xu et al., 2019). Because these strains are strictly anaerobic, anaerobic jars equipped with AnaeroPacks (Mitsubishi Gas Chemical, Tokyo, Japan) and oxygen indicators (Mitsubishi Gas Chemical, Tokyo, Japan) were used for agar plate incubation, while the anaerobic culture bottles inflated with N_2_/CO_2_ (80:20, v/v) atmosphere were employed for broth cultivation. Modified R2A broth supplemented with 10% (v/v) dimethyl sulfoxide was used for long-term storage at -80°C. In addition, for genome sequence, five type strains, *Geobacter pelophilus* DSM 12255^T^, *Geobacter grbiciae* DSM 13689^T^, *Geobacter hydrogenophilus* DSM 13691^T^, *Geobacter chapellei* DSM 13688^T^, and *Geobacter luticola* JCM 17780^T^, obtained from the German Collection of Microorganisms and Cell Cultures (DSMZ) and Japan Collection of Microorganisms (JCM), were also cultured in this study using the MFM along with 20mM acetate and 5mM Fe(III)-NTA as the electron donor and acceptor, respectively.

### 16S rRNA Gene Similarity and Phylogenetic Analysis

The nearly complete 16S rRNA gene sequences of the two isolated strains were amplified using colony PCR with the primers, 27F and 1492R, and sequenced as described previously (Kawano et al., 2020), and the 16S rRNA gene sequences of other type strains in the order *Geobacterales* were retrieved from the NCBI database unless otherwise stated. The 16S rRNA gene similarity between each pair of all studied strains was determined using the Identify service of EZBioCloud (Yoon et al., 2017). Phylogenetic trees based on the single 16S rRNA gene sequences were constructed using MEGA X software by implementing the neighbor-joining (NJ), maximum-likelihood (ML), and maximum-parsimony (MP) algorithms with 1,000 bootstrap replicates after sequences were aligned using the CLUSTAL W algorithm in MEGA X (Kumar et al., 2018). ML trees were reconstructed using the best-fit substitution model Kimura 2-parameter + G+I, while MP trees were reconstructed with the default Subtree-Pruning-Regrafting method.

### Genome Sequencing and Collection

Genomic DNA samples of the two isolated strains and five type strains were extracted by using a DNeasy Blood and Tissue Kit (Qiagen, Germany), according to the manufacturer’s instructions for Gram-stain negative bacteria. The quality and quantity of all DNA samples were determined using a NanoDrop 1000 Spectrophotometer (Thermo Fisher Scientific, United States) and Qubit 2.0 Fluorometer (Invitrogen, United States) with the corresponding reagents. Next, the DNA samples were fragmented randomly by sonication to produce DNA fragments of less than 500bp, and then end repair and adapter ligation were carried out. After amplification and purification, the qualified libraries with different indices were constructed and then sequenced using the Illumina HiSeq instrument (Illumina, United States) with a 2×150 paired-end configuration at Genewiz Inc. (Suzhou, China). The clean reads (*ca.* 2 Gbp), generated from raw reads by quality trimming, were assembled into longer contigs using the software SPAdes version 3.12.0 with default parameters (Bankevich et al., 2012), while Velvet (version 1.2.10) and SOAPdenovo (version 2.04) were adopted to assemble genomes for strains Red51^T^ and Red69^T^, respectively (Li et al., 2008; Zerbino and Birney, 2008). In addition to the genomes obtained from this study, the other reference genomes corresponding to most type species in the order *Geobacterales* were retrieved from the NCBI database with accession numbers listed in Table 1 and analyzed in parallel in the following steps.

**Table 1:** General genomic features for *Geobacterales* strains included in this study.

Strain	Genome size (Mbp)	G+C content (%)	Number of contigs	Accession numbers	Genome quality (%)	
					Completeness	Contamination
*Geomonas azotofigens* Red51^T^	5.0	62.4	23	JAHLME000000000^a^	100	0
*Geomonas diazotrophica* Red69^T^	4.7	61.9	58	JAHLMF000000000^a^	100	0
*Geomonas silvestris* Red330^T^	5.1	62.6	36	BLXX01000000	99.32	0.65
*Geomonas limicola* Red745^T^	5.2	61.8	17	BLXZ01000000	99.35	0.65
*Geomonas bemidjiensis* Bem^T^	4.6	60.3	1	CP001124.1^b^	99.78	0
*Geomonas bremensis* R1	4.7	60.0	82	AUGE01000001.1	100	0.65
*Geomonas paludis* Red736^T^	5.1	62.4	30	BLXY01000000	99.35	0.65
*Geomonas oryzae* S43^T^	4.9	61.2	18	RAHW00000000	99.35	0.65
*Geomonas edaphica* Red53^T^	4.8	60.5	17	SSYB00000000	99.35	0
*Geomonas ferrireducens* S62^T^	4.8	60.7	16	SSYA00000000	99.35	0
*Geomonas terrae* Red111^T^	4.7	61.0	8	SRSC00000000	97.42	0.65
*Geobacter pelophilus* DSM 12255^T^	4.4	53.1	42	JAHCVJ000000000^a^	99.35	0
*Geotalea uraniireducens* Rf4^T^	5.1	54.0	1	CP000698.1^b^	97.66	0
*Geotalea toluenoxydans* JCM 15764^T^	4.2	54.4	77	BBCJ01000001.1	81.98	0
*Geotalea daltonii* FRC-32^T^	4.3	53.0	1	CP001390.1^b^	99.35	0
*Geobacter luticola* JCM 17780^T^	3.7	58.2	40	JAHCVK000000000^a^	99.03	0.32
*Geobacter argillaceus* ATCC BAA-1139^T^	4.4	58.2	71	VLLN00000000.1	100	0
*Geobacter sulfurreducens* PCA^T^	3.8	60.9	1	AE017180.2^b^	99.35	0
*Geobacter anodireducens* SD-1^T^	3.7	61.5	1	CP014963.1^b^	97.85	0
*Geobacter pickeringii* G13^T^	3.6	62.3	1	CP009788.1^b^	99.84	0
*Geobacter soli* GSS01^T^	3.7	61.8	18	JXBL00000000.1	98.67	0.65
*Geobacter hydrogenophilus* DSM 13691^T^	4.0	59.6	50	JAHCZI000000000^a^	98.10	0
*Geobacter grbiciae* DSM 13689^T^	4.2	59.5	63	JAHDIW000000000^a^	98.76	0
*Geobacter metallireducens* GS-15^T^	4.0	59.8	2	CP000148.1, CP000149.1^b^	99.42	0
*Trichlorobacter lovleyi* SZ^T^	4.0	54.8	2	CP001089.1, CP001090.1^b^	99.68	0.65
*Trichlorobacter thiogenes* ATCC BAA-34^T^	3.6	52.8	44	FUWR01000042.1	100	0.65
*Oryzomonas japonica* Red96^T^	3.6	59.0	30	VZQZ00000000	99.35	0
*Oryzomonas sagensis* Red100^T^	3.6	59.7	18	VZRA00000000	99.35	0.65
*Oryzomonas rubra* Red88^T^	3.8	58.4	23	SRSD00000000	99.35	0
*Pelobacter propionicus* DSM 2379^T^	4.2	58.5	3	CP000482.1, CP000483.1, CP000484.1^b^	97.17	0.86
*Geobacter chapellei* DSM 13688^T^	3.9	51.1	60	JAHDYS000000000^a^	99.35	0

a *Genome was sequenced in this study*.

b *complete genome sequence with a circle map*.

The genome of the type strain *Geobacter pelophilus* Dfr2^T^ has been sequenced previously with the NCBI accession number BDQG00000000.1 (Aoyagi et al., 2017); however, it shared the highest similarity with the species *Geomonas bremensis* R1 (Xu et al., 2019), which was clearly distinct from the phylogenetic positions originally revealed by the 16S rRNA genes (Straub and Buchholz-cleven, 2001). Moreover, the 16S rRNA gene derived from this genome shared only 92.9% similarity with the original 16S rRNA gene (accession number: NR_026077.1) of the strain *G. pelophilus* Dfr2^T^. These conflicting findings indicate that the available genome sequence of *G. pelophilus* Dfr2^T^ is misguiding and the genome-based taxonomic position of this species also needs to be revised. Here, we re-sequenced and assembled the genome of the type strain *G. pelophilus* DSM 12255^T^, which showed 100% 16S rRNA gene similarity with the original 16S rRNA gene (NR_026077.1) of the strain *G. pelophilus* Dfr2^T^ based on the pairwise comparison; thus, the updated genome sequence (accession number: JAHCVJ000000000) of species *G. pelophilus* was used in this study.

### Multigene Based Phylogenetic Analysis

The family *Geobacteraceae* was first proposed and reclassified based on the phylogenetic analysis of the 16S rRNA gene and five housekeeping genes (*fusA*, *gyrB*, *recA*, *rpoB*, and *nifD*; Holmes et al., 2004a). Thus, a phylogenetic tree of multilocus sequence analysis (MLSA) based on the four concatenated housekeeping genes (*fusA*, *gyrB*, *recA*, and *rpoB*) was constructed. The *nifD* gene was excluded from this study because of its absence in some species of the order *Geobacterales*. The amino acid sequences of the four housekeeping genes were retrieved from the respective genomes using the BLASTP tool of the local BLAST server (Camacho et al., 2009) with the query sequences from the genome-annotated strain *Geobacter metallireducens* GS-15T. Sequence alignment, concatenation, and tree construction were then performed using CLUSTAL W and MEGA X software with the ML algorithm based upon the best-fit substitution model (Kumar et al., 2018). In addition, the whole genome-based phylogeny was reported to be more robust and reproducible and was recently encouraged for prokaryotic taxonomy owing to the rapid development of high-throughput sequencing technologies and more available genomes (Chun et al., 2018). Thus, two phylogenomic trees were also constructed using the Genome Taxonomy Database Toolkit (GTDB-Tk, version 0.1.3) and up-to-date bacterial core gene set (UBCG) pipelines, respectively, with default parameters as described previously (Na et al., 2018; Chaumeil et al., 2020). The bac120 tree, constructed by GTDB-Tk with the FastTree tool, was inferred from a concatenated alignment of 120 ubiquitous single-copy proteins, whereas the UBCG tree was constructed using the RAxML tool based on the amino acid sequences of 92 concatenated core genes retrieved from the analyzed genomes. Bootstrap values of both phylogenomic trees were evaluated based on 100 replicates. All trees were further polished and visualized using the interactive tree of life (iTOL) v5 (Letunic and Bork, 2021).

### Genome Annotation and Comparison

As most of the genomes analyzed in this study were in draft status, the genome quality was first assessed for completeness and contamination using CheckM version 1.0.18, with the default parameters (Parks et al., 2015). Open reading frame prediction and functional gene annotation were performed based on the SEED database using the RAST server with the ClassicRAST annotation scheme (Aziz et al., 2008) and the NCBI Refseq database using the NCBI Prokaryotic Genome Annotation Pipeline (Pruitt et al., 2005). The metabolic pathways were annotated with the Kyoto Encyclopedia of Genes and Genomes database (Kanehisa and Goto, 2000). The DNA G+C content of all analyzed strains was calculated from the genome sequences as described by Meier-Kolthoff et al. (2014). The genome similarities between each pair of *Geobacterales* strains were quantified by the average nucleotide identity (ANI) at the nucleotide level, as well as the average amino acid identity (AAI) and percentage of conserved protein (POCP), at the amino acid level. The ANI values between each pair of strains were calculated *in silico* using the JSpeciesWS tool with the BLAST+ algorithm (Richter et al., 2016), whereas AAI and POCP values between each pair of strains were determined using the AAI calculator web server of Kostas lab^2^ (Konstantinidis and Tiedje, 2005) and a Python script^3^ based on the formula as described previously (Qin et al., 2014), respectively. Dendrograms based on the pairwise AAI and POCP values were constructed and visualized using the bactaxR package of R version 4.0.4 (Carroll et al., 2020). The visualization of the numerical correlation and matrix for ANI, AAI, and POCP values was carried out by using the packages ggplot2, pheatmap, and complexHeatmap in R version 4.0.4 (Wickham, 2011; Kolde and Kolde, 2015; Gu et al., 2016).

### Phenotypic Characterization

Cell morphology, including bacterial shape and size, was observed using transmission electron microscopy (TEM, model JEM-1400, JEOL, Tokyo, Japan) after cells were cultured on modified R2A agar for 5days and negatively stained with ammonium molybdate. Gram staining reaction was performed using a commercial Gram-staining Kit (Sigma, St Louis, MO, United States) according to the manufacturer’s instructions. Colony morphology was observed on modified R2A agar plates after incubation for 5days. The temperature range and optimum temperature for bacterial growth were examined on modified R2A agar plates and in modified R2A broth at 10, 13, 16, 20, 25, 30, 33, 37, 40, and 45°C. The pH ranges and optimum pH for growth were assayed between 5.0 and 9.0 (in increments of 0.5 pH units) in modified R2A broth gassed with He at 30°C supplemented with 20mM pH buffer solutions as described previously (Itoh et al., 2021). Bacterial growth on the broth conditions was quantified using a spectrophotometer (Jasco V550, Tokyo, Japan) at 600nm wavelength (OD_600_) after 3days of incubation. The requirement and tolerance to NaCl concentration of bacterial growth were examined on modified R2A agar plates in the presence of 0–1.0% (w/v) NaCl at intervals of 0.1%. The bacterial mean generation time was calculated according to Todar’s Online Textbook of Bacteriology,^4^ and the growth curves were generated based on the OD_600_ values of biomass over time, using 50ml modified R2A broth in 100ml serum bottles (N_2_/CO_2_) at 30°C with 1/100 inoculation scale (Supplementary Figure 1). Electron acceptor and donor utilization tests were carried out as described previously using MFM with Fe(III)-NTA (10mM) as the electron acceptor for all electron donor tests and acetate (10mM) as the electron donor for all electron acceptor tests (Itoh et al., 2021). The final concentrations of the tested electron donors and acceptors were the same as those described by Nevin et al. (2005). Cytochrome *c* analysis was performed using the whole cells anaerobically cultured in MFM with 20mM acetate and 10mM fumarate. The final absorption spectra were generated using the absorption spectra of dithionite (20μM)-reduced cells minus the absorption spectra of air-oxidized cells using a Jasco V550 spectrophotometer. Nitrogen-fixation capabilities of the two strains were confirmed by bacterial growth under optimal conditions using MFM (excluding NH_4_Cl) with N_2_ (80% of the atmosphere) as the only nitrogen source. Growth conditions were determined using a Coulter counter (Multisizer 4e, Beckman Couter Inc., United States) over time. The activity of constitutive enzymes was determined using the API ZYM strips (bioMérieux, France) at 30°C for 4h, in accordance with the manufacturer’s instructions, while the catalase activity was assayed by the formation of gas bubbles after bacterial cells on the plates were immersed in H_2_O_2_ (3%, v/v) solution at room temperature.

### Chemotaxonomic Characterization

For cellular fatty acid profile analysis, the three strains together with the reference strain *Geomonas oryzae* S43^T^ were cultured in modified R2A broth at 30°C for 3days until the bacteria were in the late exponential growth phase, and then spun down for biomass collection. Fatty acids were extracted from the freeze-dried biomass according to a previously described method (Kuykendall et al., 1988) and detected using the Sherlock Microbial Identification System (version 6.0) with the MOORE 6 database. Respiratory quinone profiles of the three strains were determined by using high-performance liquid chromatography (HPLC) with an ACQUITY UPLC H-Class system (Waters, United States) with the biomass cultured in modified R2A broth at 30°C for 5days. The profile analysis of both fatty acids and respiratory quinones was carried out by TechnoSuruga Laboratory Co., Ltd. (Shizuoka, Japan).

## Results and Discussion

### 16S rRNA Gene Similarity and Phylogeny

The 16S rRNA gene-based pairwise comparisons of all type strains in the genus *Geomonas* showed 96.4–99.8% similarity values at the intra-genus level and showed a maximum similarity of 96.9% to the species in the other genera (Supplementary Figure 2). The two isolated strains, Red51^T^ and Red69^T^, shared the highest similarities with *G. paludis* Red736^T^ (98.5%) and *G. oryzae* S43^T^ (98.6%), respectively, suggesting that these two strains may be two novel members in the genus *Geomonas*. Moreover, in the genus *Geobacter*, the 16S rRNA gene-based pairwise comparisons showed 94.3–99.6% similarity values at the intra-genus level, and in the family ‘*Pseudopelobacteraceae*’, the two species in the genus *Trichlorobacter* shared 98.6% 16S rRNA gene similarity and showed a maximum similarity of 95.7% to the species in the other genera (Supplementary Figure 2). In general, the values 94.5–95.0% and 98.65% of 16S rRNA gene identities are used as taxonomic thresholds for genus and species separation, respectively (Kim et al., 2014; Yarza et al., 2014). Therefore, our findings revealed that few species in the order *Geobacterales* strictly respected the recommended genus and species boundary values of 16S rRNA gene identity for bacterial affiliation, which indicated that the common taxonomic criteria based on 16S rRNA gene similarity are inappropriate for delineating the *Geobacterales* species, as reported previously for the genus *Mycobacterium* and many of the human-associated genera and species (Rossi-Tamisier et al., 2015; Beye et al., 2018).

**Figure 1:**
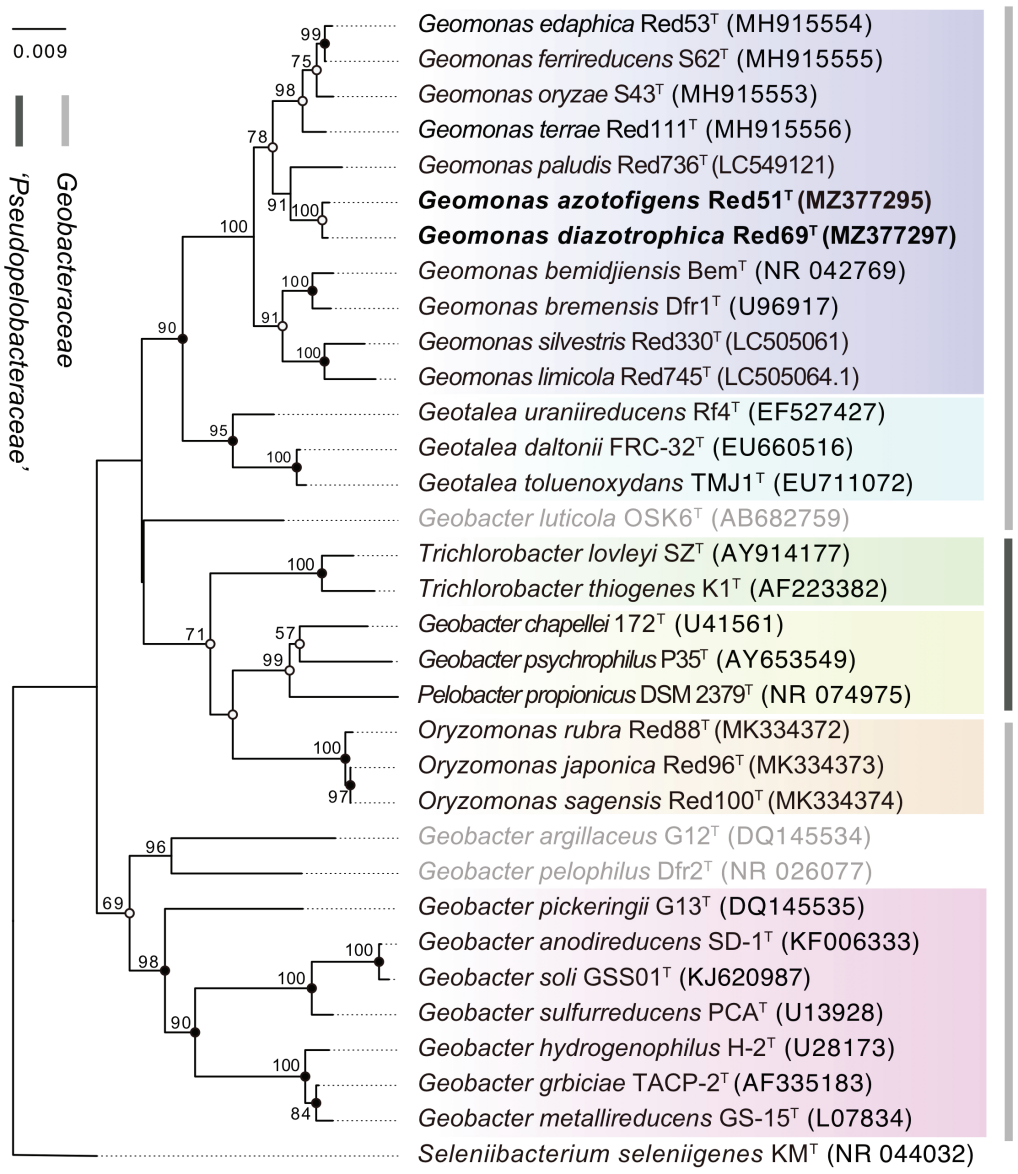
Phylogenetic tree of species in the order *Geobacterales* based on 16S rRNA sequence divergence. The tree was inferred by the neighbor-joining (NJ) algorithm using MEGA X with Kimura 2-parameter model. Filled circles indicate that the corresponding nodes were also recovered in ML and MP algorithm. Open circles indicate that the corresponding nodes were also recovered in either ML or MP algorithm. The three species with pendent taxonomic positions are marked in grey. The background colors represent different bacterial genera, and yellow color represents the genus ‘*Pseudopelobacter*’. Bootstrap values (expressed as percentages of 1,000 replications) over 50% are shown at branching nodes. Bar, 0.009 substitutions per nucleotide position.

Moreover, in the 16S rRNA gene-based phylogenetic tree, the type strains in the same genus were mostly clustered together into independent branches and clearly separated from their phylogenetic neighbors, suggesting that the two isolated strains Red51^T^ and Red69^T^, robustly located in the cluster of *Geomonas* species, phylogenetically belong to the genus *Geomonas* and share their close relationships with *G. paludis* Red736^T^ (Figure 1). However, three species, *Geobacter luticola* OSK6^T^, *Geobacter argillaceus* G12^T^, and *Geobacter pelophilus* Dfr2^T^, showed variability, as each was located in an independent branch that was different from their nominal groups in the phylogenetic tree, indicating that these three strains should be allocated to higher taxonomic ranks. The order *Geobacterales* currently contains two families, *Geobacteraceae* and ‘*Pseudopelobacteraceae*’, which should represent two independent monophyletic branches in the phylogenetic tree. However, the real tree structure based on the 16S rRNA gene placed the two genera *Trichlorobacter* and ‘*Pseudopelobacter*’ in the family ‘*Pseudopelobacteraceae*’ into the family *Geobacteraceae* and formed an unstable branch with the genera *Geomonas* and *Geotalea* (Figure 1). This conflicting tree structure suggests a relatively low phylogenetic resolution and unstable tree topology at the family level based on the single 16S rRNA gene; thus, additional phylogenetic analysis based on multigene is required.

### Multigene-Based Phylogenetic Analysis

**Figure 2:**
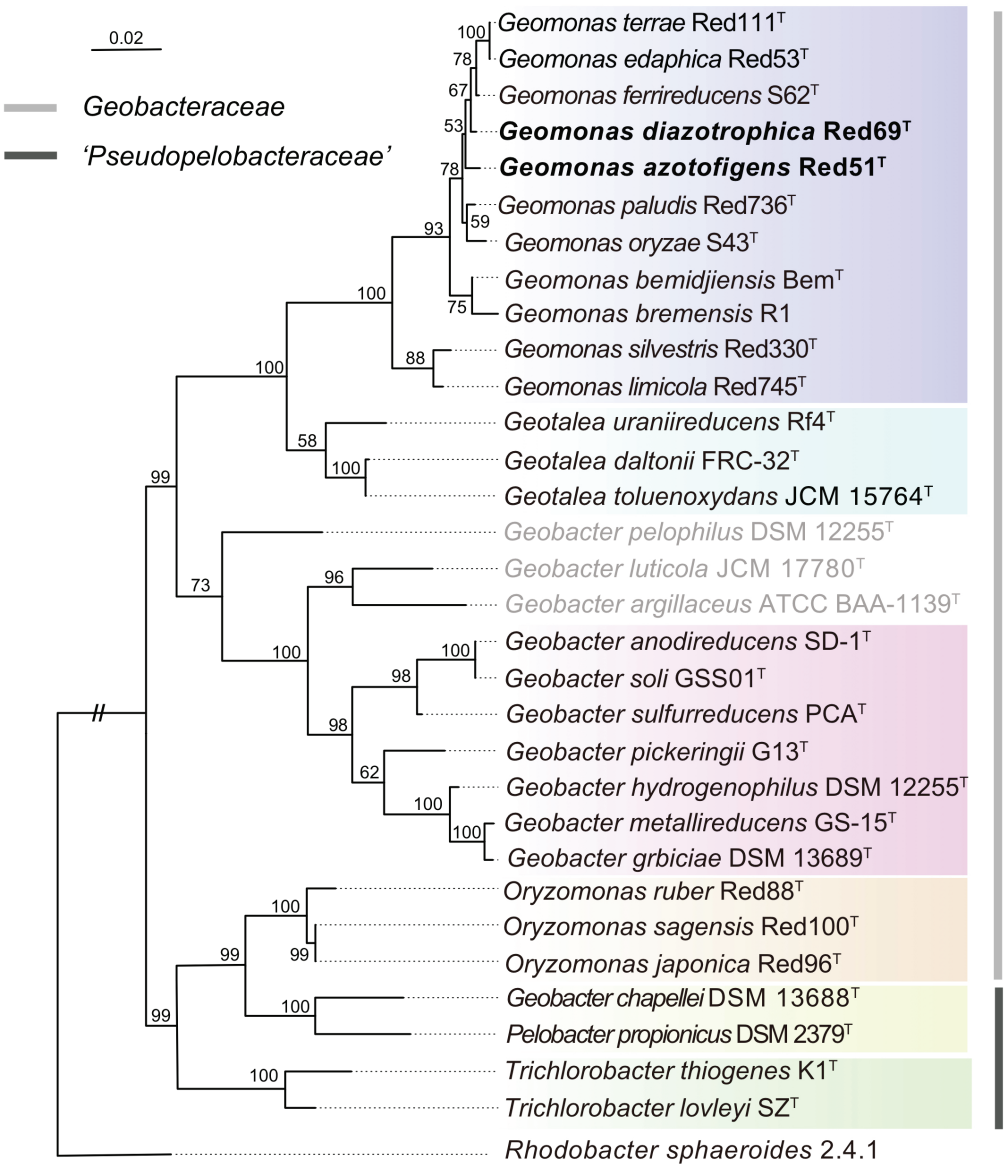
Maximum-likelihood (ML) phylogenetic tree of species in the order *Geobacterales* based on the multilocus sequence analysis. This tree was constructed based on the deduced amino acid sequences of the four concatenated housekeeping gene: *fusA* (1–196 amino acids), *gyrB* (197–491 amino acids), *recA* (492–716 amino acids), and *rpoB* (717–916 amino acids), using MEGA X with LG+G model. The three species with pendent taxonomic positions are marked in grey. The background colors represent different bacterial genera, and yellow color represents the genus ‘*Pseudopelobacter*’. Bootstrap values (expressed as percentages of 500 replications) over 50% are shown at branching nodes. Bar, 0.02 substitutions per amino acid position.

**Figure 3:**
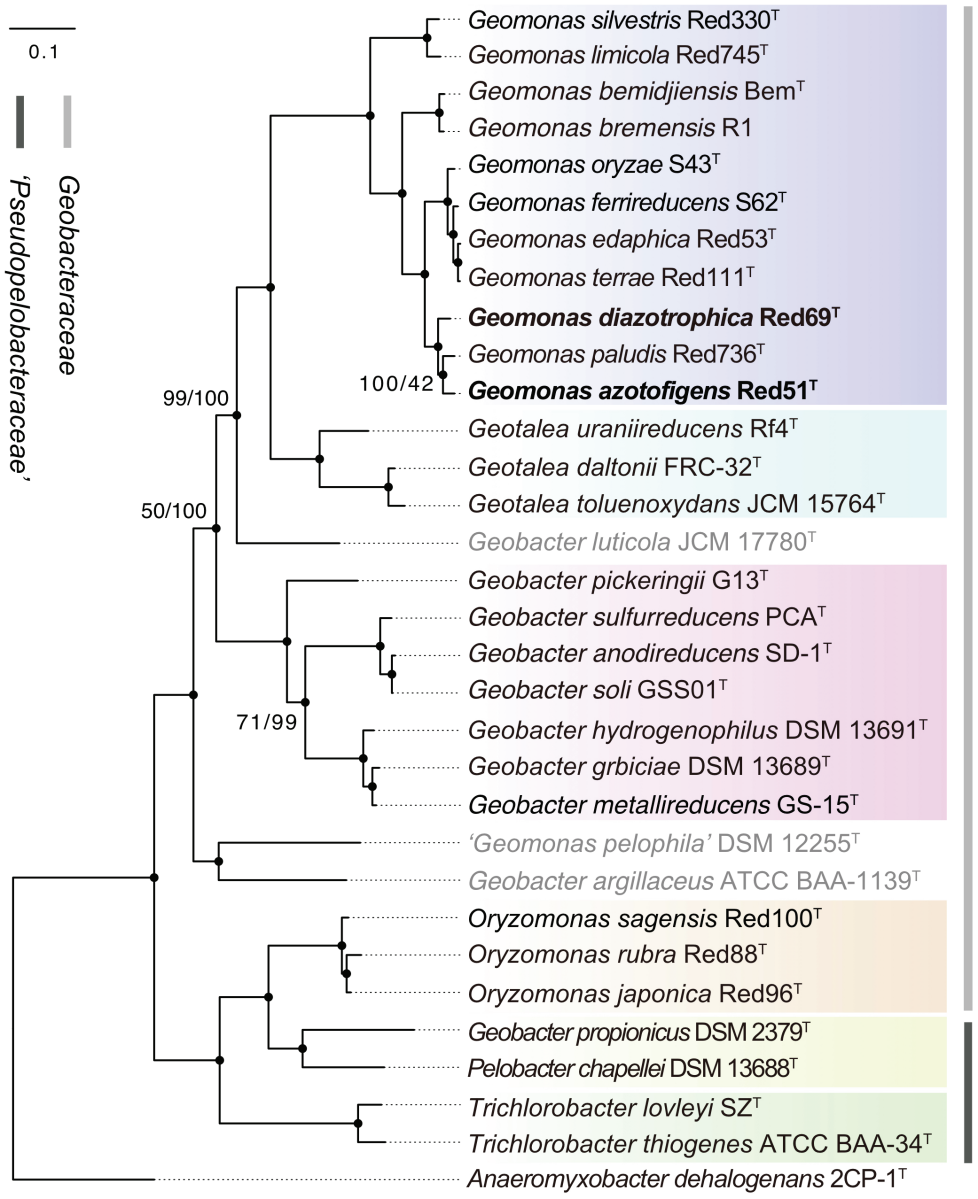
Maximum-likelihood (ML) phylogenomic tree of species in the order *Geobacterales* based on a concatenated alignment of 120 ubiquitous single-copy proteins. This tree was inferred by GTDB-Tk pipeline equipped with FastTree tool with the WAG+CAT model. Filled circles indicate that the corresponding nodes were also recovered in up-to-date bacterial core gene (UBCG) tree. The three species with pendent taxonomic positions are marked in grey. The background colors represent different bacterial genera, and yellow color represents the genus ‘*Pseudopelobacter*’. Bootstrap values, at least one less than 100% for GTDB/UBCG trees based on 100 replications, are shown at branching nodes. Bar, 0.1 substitutions per amino acid position.

To remedy the low resolution of the phylogenetic analysis based on 16S rRNA genes, we undertook phylogenetic analysis based on multiple concatenated genes. Firstly, an MLSA tree based on four concatenated housekeeping genes (*fusA*, *gyrB*, *recA*, and *rpoB*) was constructed, which clearly separated the two families *Geobacteraceae* and ‘*Pseudopelobacteraceae*’ into two independent monophyletic branches (Figure 2). Next, two phylogenomic trees, based on the concatenated 120 ubiquitous single-copy proteins and 92 core genes using GTDB and UBCG pipelines, were constructed to show the genomic evolutionary distance of the species in the order *Geobacterales* (Figure 3). These two phylogenomic trees showed topologies identical to each other and shared a similar tree structure to the MLSA tree, although with distinct but high bootstrap values at tree branches when using different tree-constructing sequences, which suggested the robust phylogenomic status of the species in the order *Geobacterales*. Therefore, the genome-based phylogenies were adopted as the primary guideline for the reassignment of the species in the order *Geobacterales*; this is consistent with the previous reclassifying proposal of the order *Geobacterales* that the phylogenomic analysis be mainly employed as the taxonomic criterion (Waite et al., 2020). Given the coherent, stable, and monophyletic positions on the multigene-based phylogenetic trees and the criterion that one genus should be clustered into only one phylogenetic group, most species in the three genera *Geomonas*, *Geotalea*, and *Geobacter* were clearly separated from their close genera in the family *Geobacteraceae* and rightly placed without any taxonomic controversy, including the two novel strains Red51^T^ and Red69^T^, which were robustly located in the *Geomonas* group (Figures 2, 3). However, there was an exception for the three species, *G. luticola*, *G. argillaceus*, and *G. pelophilus*, which were treated as taxonomy-pendent species owing to their absent or wrong genome sequences but were also phylogenomically assigned to the cluster of the family *Geobacteraceae* based on their updated genome sequences (Figure 3). The position of species *G. luticola* was independently located between the two clusters of the genera *Geobacter* and *Geotalea*/*Geomonas* with bootstrap values above 95% (Figure 3), which indicated that the sole species *G. luticola* is a novel genus in the family *Geobacteraceae*. Similarly, the other two species *G. argillaceus* and *G. pelophilus*, that formed a coherent branch, were placed as the outermost species of the family *Geobacteraceae* in the phylogenomic trees and showed a distant evolutionary distance from the other *Geobacteraceae* members, implying that these two species consisted of a genus-level group in the family *Geobacteraceae* (Figure 3). However, on the MLSA tree, the species *G. argillaceus*, along with *G. luticola* formed a robust branch with a high bootstrap value of 96% and was clearly separated from the other species *G. pelophilus* (Figure 2). These inconsistent phylogenetic positions imply that these two taxonomy-pendent species are phylogenetically independent and may represent two different genera in the family *Geobacteraceae*. Moreover, the genus *Oryzomonas* was recently proposed with three novel species (*O. japonica*, *O. sagensis*, and *O. rubra*) as the members of the family *Geobacteraceae* (Xu et al., 2020), but all the phylogenetic trees placed this genus into the branch of the family ‘*Pseudopelobacteraceae*’ with high bootstrap values (> 70%); thus, the taxonomic status of the genus *Oryzomonas* should be reassigned into the family ‘*Pseudopelobacteraceae*’.

### Genome Characteristics

**Figure 4:**
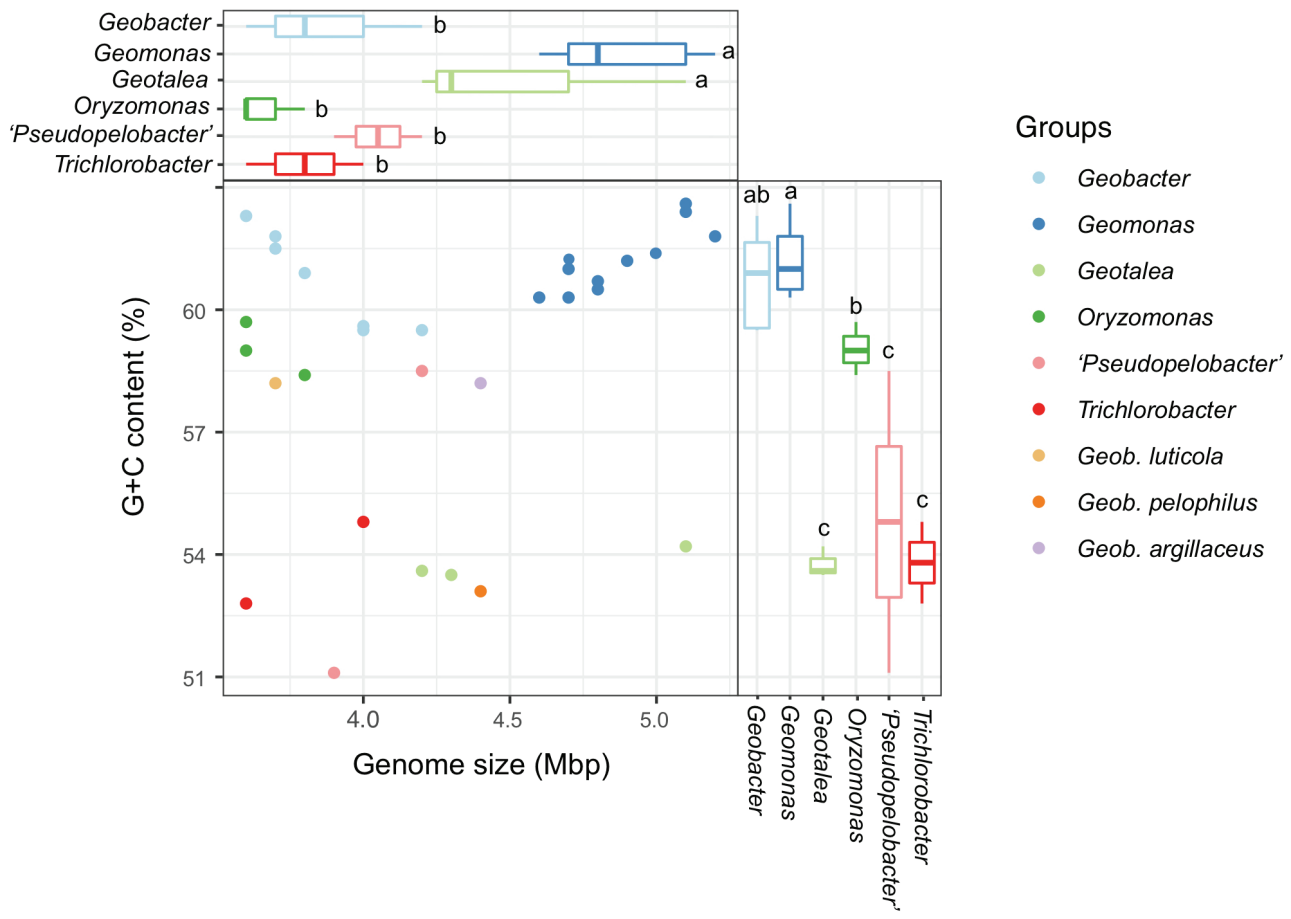
Comparison of genome size and G+C content of all species in the order *Geobacterales*. The values of G+C content were calculated based on the whole genome. The compared genera containing at least two species were selected for global statistical analysis. Different letters on the boxplots indicate significant differences (*p*<0.05) among different genera based on a one-way ANOVA followed by LSD significant difference test.

In this study, a total of 31 bacterial genomes, corresponding to the 31 reference species in the order *Geobacterales*, were obtained and analyzed in parallel (Table 1). Eight of them were completely assembled with circle maps, whereas the others were draft genomes with multiple contigs and nucleic acid gaps. Moreover, most of them were determined to be near-complete genomes with high genomic completeness (>97%) and low genomic contamination (<1.0%), except for strain *Geotalea toluenoxydans* JCM15764^T^, whose genomic completeness was only 82.0%. The genome size among these genomes ranged from 3.6 to 5.5 Mbp with a genomic G+C content of 51.1–62.6%. In detail, the genome size in the six genera *Geomonas*, *Geotalea*, *Trichlorobacter*, *Oryzomonas*, ‘*Pseudopelobacter*’, and *Geobacter* was 4.6–5.5 Mbp, 4.2–5.1 Mbp, 3.6–4.0 Mbp, 3.6–3.8 Mbp, 3.9–4.2 Mbp, and 3.6–5.1 Mbp, with the genomic G+C content of 60.3–62.6%, 53.5–54.2%, 52.8–54.8%, 58.4–59.7%, 51.1–63.8%, and 59.5–62.3%, respectively, whereas for the three species with pendent taxonomic statuses, *G. luticola*, *G. Argillaceus,* and *G. pelophilus*, their genome sizes were 3.7 Mbp, 4.4 Mbp, and 4.4 Mbp with the genomic G+C content of 58.2%, 58.2%, and 53.1%, respectively (Table 1; Figure 4). Based on the variation in these two genome features, the four genera *Geomonas*, *Geotalea*, *Trichlorobacter*, and *Geobacter* were clearly separated from each other (ANOVA with LSD test, *p*<0.05; Figure 4), indicating the conserved genomic characteristics among the different genera in the order *Geobacterales*, which further confirmed that the two novel strains Red51^T^ and Red69^T^ are two members of the genus *Geomonas*, as their genome sizes (5.0 and 4.7 Mbp) and G+C content (62.4% and 61.9%) only met the range of this genus. Furthermore, Meier-Kolthoff et al. (2014) noted that the within species variation in DNA G+C content is at most 5% based on hundreds of bacterial species analysis, which indicated that the three species in the genus ‘*Pseudopelobacter*’ with a genomic G+C content variation of more than 5% among each other may be monophyletic and represent three different genera (Table 1; Figure 4). The species *G. pelophilus* was clustered into the *Geomonas* group based on the old genome sequence (BDQG00000000.1; Xu et al., 2019; Waite et al., 2020), but the updated genome (JAHCVJ000000000) of this species showed a genomic G+C content of 53.1% and genome size of 4.4 Mbp, which are much smaller than those of the species in the genus *Geomonas* (60.3–62.6% for G+C content and 4.6–5.5 Mbp for genome size; Table 1; Figure 4), implying that the real taxonomic position of this species would not be clustered into the genus *Geomonas*. Similarly, the other two taxonomy-pendent species, *G. luticola* and *G. argillaceus*, were also excluded from the range of their original genus *Geobacter* according to these two genome features, further supporting the revision of the current taxonomic positions of these two species.

In addition, based on the genomic annotation, the two isolated strains Red51^T^ and Red69^T^ were both found to possess a complete nitrogen fixing pathway with multiple related genes in the construction of Mo-type nitrogenase (*nifHDK*), and biosynthesis of FeMo cofactor (*nifBENX*; Supplementary Table 1), which is consistent with their phenotypic features that they grow well with N_2_ as the sole nitrogen source. Notably, distinct from the strain Red69^T^, strain Red51^T^ also harbors a *vnfEN* gene cluster, which is part of the V-type nitrogenase pathway (Supplementary Table 1). Furthermore, each of the two strains was annotated with a complete assimilatory sulfate reduction process (Supplementary Table 1), in which sulfate can be reduced to H_2_S. Along with their ability to reduce iron, these two strains extensively participate in multiple biogeochemical processes in paddy soils, suggesting their great potential as representative bacteria to investigate microbe-mediated environmental challenges.

### Whole-Genome Similarity Indices

**Figure 5:**
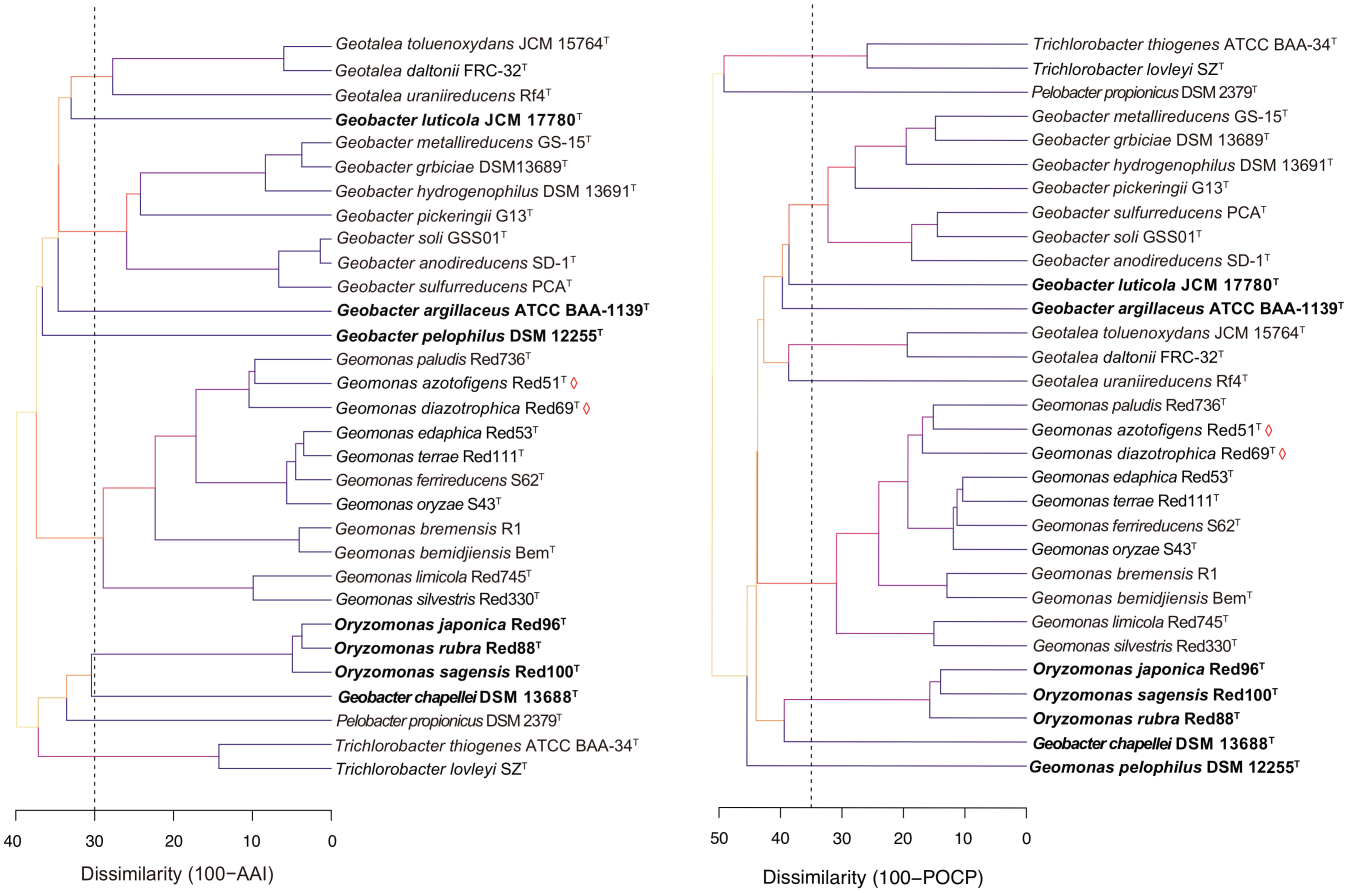
Tree cladograms of species in the order *Geobacterales* based on the genome similarity indexes, amino acid identity (AAI; left) and percentage of conserved protein (POCP; right). Branch colors on the trees correspond to different dissimilarity values (%). Dashed vertical lines, appeared at dissimilarities of 30 of AAI and 35 of POCP, correspond to AAI and POCP thresholds of 70% and 65%, respectively. Bacterial names labeled by red diamonds indicated the two novel isolated species. The species should be taxonomically revised are in bold.

Genome similarity has been reported to show reliable and reproducible features as the numerical thresholds for the delineation of bacterial taxa and has been widely used in bacterial taxonomy (Konstantinidis and Tiedje, 2005; Richter and Rosselló-Móra, 2009; Qin et al., 2014). Here, three genomic similarity indices, including AAI and POCP based on the amino acid sequence and ANI based on the nucleic acid sequence, were also calculated to determine the similarity thresholds for taxonomic reassignment of the order *Geobacterales*. In the family *Geobacteraceae*, the different species except for the three taxonomy-pendent species shared 61.3–67.7% of AAI, 50.9–63.7% of POCP, and 69.0–73.6% of ANI at inter-genus levels and shared 70.9–98.6% of AAI, 58.5–89.6% of POCP, and 73.6–99.4% of ANI at intra-genus levels; whereas in the family ‘*Pseudopelobacteraceae*’ (including *Oryzomonas*), the strains shared 61.1–69.7% of AAI, 50.5–61.4% of POCP, and 69.1–74.0% of ANI at the inter-genus level and 66.3–96.3% of AAI, 50.2–86.0% of POCP, and 70.8–95.6% of ANI at intra-genus level (Supplementary Figures 3, 4). Because genomic relatedness with amino acid sequences was more recommended for genera separation (Qin et al., 2014), AAI and POCP were primarily evaluated with the threshold values of 70% and 65%, respectively, as proposed for the genera *Geomonas* and *Oryzomonas* in our previous studies (Xu et al., 2019, 2020). As shown in Figure 5, the cut-off value of 70% for AAI clustered the five genera, *Geomonas*, *Geotalea*, *Geobacter*, *Trichlorobacter*, and *Oryzomonas* into independent branches clearly, whereas the POCP value of 65% clearly separated the four genera *Geomonas*, *Geobacter*, *Trichlorobacter*, and *Oryzomonas*. Similar to the phylogenomic tree as mentioned above, these two threshold values of the genomic similarity indexes at the amino acid level also contribute equally to the taxonomy of the species in the order *Geobacterales*, which is in line with the previous reports for several families, such as *Methylococcaceae*, *Methylothermaceae*, and *Rhodobacteraceae*, using the genomic similarity index as the boundaries for genera delineation (Skennerton et al., 2015; Orata et al., 2018; Wirth and Whitman, 2018). Notably, both genome-similarity-based trees placed the two members (*Pelobacter propionicus* and *Geobacter chapellei*) of the genus ‘*Pseudopelobacter*’ into different branches (Figure 5), revealing their low genome identity to each other, which suggested the same proposal as the genomic analysis that these two species should be reassigned into two independent genera. Moreover, the three taxonomy-pendent species formed three independent branches with low genome identity to any other close species in the genome-similarity-based trees, further confirming the genus levels of these three species in the taxonomy. Although the POCP values of 65% also separated the genus *Geotalea* into two branches on the POCP-based tree, there was no obvious difference in the genome characteristics observed within the three *Geotalea* species, and the phylogenomic analysis and AAI values also did not support their separation. Thus, an AAI of 70% was more recommended as the taxonomic threshold for the genus *Geotalea*.

**Figure 6:**
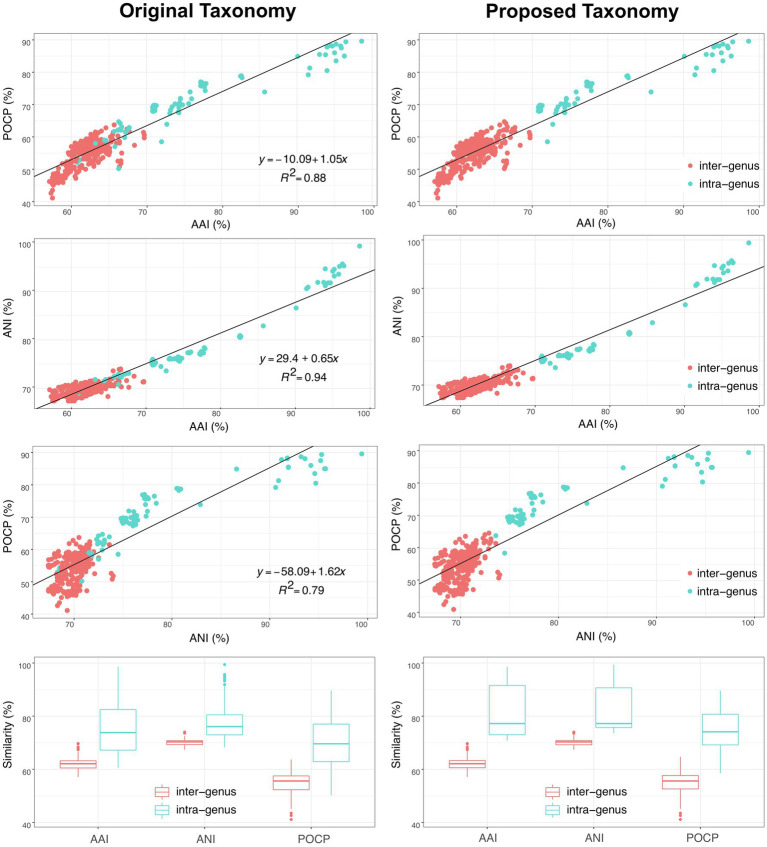
Comparison of original (left) and proposed (right) taxonomy of bacteria within the order *Geobacterales* based on three different genomic similarity indexes (AAI, ANI and POCP). Red and blue dots/boxes indicate inter-genus and intra-genus, respectively.

**Figure 7:**
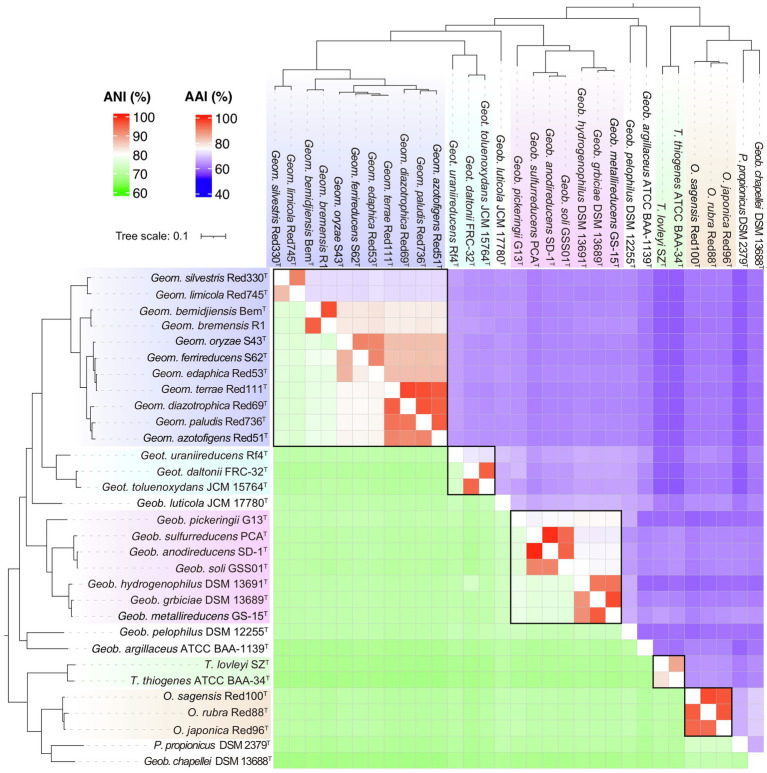
Relationship of phylogeny and similarity based on the whole genome sequences of bacteria in the order *Geobacterales*. The heatmap consists of AAI values in upper-right and ANI values in lower-left. The phylogenomic tree was constructed using the GTDB-Tk pipeline. The bacteria in the different proposed genera are shown in different colors.

In addition, another genomic identity index based on the nucleic acid sequence identity, ANI, was also evaluated as a taxonomic criterion in the order *Geobacterales*. As shown in Figure 6, besides the linear relationships between AAI and POCP with a correlation coefficient (*R*^2^) of 0.89, ANI also showed a linear relationship with AAI and POCP values and shared the highest correlation coefficient (*R*^2^=0.94) with AAI, indicating that ANI can also be a criterion for genera separation in the order *Geobacterales*. Based on the parting lines between intra- and inter-genus, the threshold of ANI for genera separation was approximately 74%, which is almost the same as the reported genus demarcation boundary of ANI with a mean value of 73.98% and a median value of 73.11% from an analysis of hundreds of genera (Barco et al., 2020). Furthermore, a matrix heatmap based on the AAI and ANI values along with the phylogenomic tree is shown in Figure 7. The circled grids with similar colors of the heatmap representing close bacterial species are well mapped to the independent branches on the phylogenomic trees, suggesting a complementary relationship between phylogenetic analysis and genomic similarity indices across the species in the order *Geobacterales*.

The two novel strains Red51^T^ and Red69^T^ share the highest genome similarities to the type strains in the genus *Geomonas* with values of 75.7–87.5% for ANI, 71.1–90.3% for AAI, and 68.7–84.8% for POCP (Supplementary Figures 3, 4), which are all higher than the proposed thresholds for genus separation as described above, suggesting the affiliation of these two strains in the genus *Geomonas*. Moreover, the common thresholds for species delineation are recommended as 95–96% for ANI and 95% for AAI (Luo et al., 2014; Chun et al., 2018), these two novel strains, thus, represent two different species in the genus *Geomonas*.

### Phenotypic and Chemotaxonomic Characteristics of the Two Isolated Strains

Cells of the two isolated strains, Red51^T^ and Red69^T^, were identified as Gram-stain negative, strictly anaerobic, motile, and rod-shaped. TEM images showed that these two strains were 0.5–0.8μm wide and 1.0–2.8μm long in cellular size and contained peritrichous flagella for their motility (Supplementary Figure 5). The colonies on the modified R2A agar plates were observed as red or light red, circular, smooth, and approximately 1.0mM in diameter after 5days of incubation at 30°C. Growth conditions of these two strains were commonly occurred at 15–42°C (optimum, 30–33°C), pH 5.5–8.0 (optimum, pH 6.5–7.0), and with 0–0.6% (w/v) NaCl (optimum, 0–0.2%). In addition, strain Red51^T^ could also grow at 13°C, whereas strain Red69^T^ could tolerate 0.8% (w/v) NaCl. The two strains were also found to grow with N_2_ as the sole nitrogen source, and their growth curves were similar to those with ${\mathrm{NH}}_{4}^{+}$ as the nitrogen source, suggesting that these two strains possess nitrogen-fixing ability (Supplementary Figure 6). Cytochrome *c* analysis revealed that the absorbance peaks of strain Red51^T^ were 426, 524, and 554nm, whereas those of strain Red69^T^ were 424, 524, and 554nm (Supplementary Figure 7). These different features demonstrated that these two strains were not from the same colony and represented two independent isolates. Moreover, these two strains were also found to contain esterase (C4), esterase lipase (C8), and leucine arylamidase activities, in line with many type strains in the genus *Geomonas*. Other phenotypic characteristics of these two novel strains are presented in Table 2 and the species descriptions.

**Table 2:** Differential characteristics of the two novel strains Red51^T^ and Red69^T^ and the other type strains in the genus *Geomonas*.

Characteristics	1	2	3	4	5	6	7	8	9	10	11
Isolation source	Paddy soil	Paddy soil	Paddy soil	Paddy soil	Paddy soil	Paddy soil	Paddy soil	Forest soil	Paddy soil	River sediment	Freshwater ditch
Growth temperature (°C)											
Range	13–42	15–42	20–42	13–42	10–42	13–42	10–42	20–40	20–40	15–37	ND
Optimum	30–33	30–33	30–33	30–33	30–33	30–33	30–33	30–33	30–33	30	30–32
Growth salinity (% NaCl)
Range	0–0.6	0–0.8	0–0.8	0–0.7	0–0.7	0–0.7	0–0.7	0–0.4	0–0.4	0–0.8	ND
Optimum	0–0.2	0–0.2	0–0.2	0–0.2	0–0.2	0–0.2	0–0.2	0–0.1	0–0.1	0–0.2	ND
Growth pH											
Range	5.5–8.0	5.5–8.0	5.5–7.5	5.5–8.0	5.5–8.0	5.5–8.0	5.5–8.0	5.0–7.5	5.0–7.5	5.0–7.5	ND
Optimum	6.5–7.0	6.5–7.0	6.0–7.0	6.0–7.0	6.5–7.0	6.0–7.0	6.5–7.0	6.5–7.0	6.0–6.5	6.0–6.5	5.5–6.7
Motility	+	+	+	+	+	+	+	+	+	−	−
Electron donor											
Methanol	+	+	−	+	−	+	+	−	−	−	ND
Succinate	+	+	+	+	+	+	+	−	+	+	+
Isopropanol	+	+	+	+	+	+	+	+	−	+	ND
Serine	+	+	+	+	+	+	+	−	−	+	+
Butanol	−	−	−	−	−	−	−	−	−	−	+
Electron acceptor											
Nitrate	+	+	+	+	+	+	+	+	+	−	ND
MnO_2_	−	−	−	−	−	−	−	−	−	+	+
Enzymatic activities											
Alkaline phosphatase	−	−	−	−	+	−	−	+	+	−	ND
Esterase(C4)	+	+	+	+	+	+	−	+	+	+	ND
Esterase lipase (C8)	+	+	+	+	+	+	−	+	+	+	ND
Absorbance peaks of cytochrome c (nm)	426, 524, 554	424, 524, 554	424, 525, 553	424, 524, 554	424, 522, 554	424, 524, 554	424, 524, 554	424, 524, 554	424, 524, 553	422, 522, 555	423, 523, 552

Strains: 1, *Geomonas azotofigens* Red51^T^ (this study); 2, *Geomonas diazotrophica* Red69^T^ (this study); 3, *Geomonas paludis* Red736^T^ (Itoh et al., 2021); 4, *Geomonas oryzae* S43^T^ (Xu et al., 2019); 5, *Geomonas edaphica* Red53^T^ (Xu et al., 2019); 6, *Geomonas ferrireducens* S62^T^ (Xu et al., 2019); 7, *Geomonas terrae* Red111^T^ (Xu et al., 2019); 8, *Geomonas silvestris* Red330^T^ (Itoh et al., 2021); 9, *Geomonas limicola* Red745^T^ (Itoh et al., 2021); 10, *Geomonas bemidjiensis* Bem^T^ (Nevin et al., 2005; Xu et al., 2019); 11, *Geomonas bremensis* Dfr1^T^ (Straub and Buchholz-Cleven, 2001). +, Positive; −, negative. ND, no data.

In addition, other chemotaxonomic features, such as fatty acid profiles, clearly distinguished these two strains; for example, strain Red51^T^ contained iso-C_15:0_ (10.8%), C_15:1_ *ω6c* (18.7%), and C_15:0_ (21.6%) as the major components (>10%) of fatty acids, whereas strain Red69^T^ had iso-C_15:0_ (41.9%) and C_16:1_ *ω7c* (16.2%) as the major components (Table 3). Moreover, in contrast to strain Red51^T^, strain Red69^T^ showed more similar fatty acid profiles with the type species *G. oryzae* S43^T^, because they both contain iso-C_15:0_, C_16:1_ *ω7c*, and iso-C_15:0_ 3-OH as the top three fatty acids (Table 3). Menaquinone-8 (MK-8) was characterized as the predominant quinone for the two novel strains, which is consistent with other type strains in the genus *Geomonas* (Xu et al., 2019; Itoh et al., 2021).

**Table 3:** Fatty acid compositions (%) of the two novel strains *Geomonas azotofigens* Red51^T^, *Geomonas diazotrophica* Red69T and the reference strain *Geomonas oryzae* S43T.

Fatty acids	Red51^T^	Red69^T^	S43^T^
iso-C_13:0_	0.1	0.3	1.6
iso-C_14:0_	1.3	1.4	0.5
C_14:0_	3.2	3.0	2.0
C_14:0_ DMA	0.3	0.3	1.4
C_15:0_ iso	**10.8**	**41.9**	**54.7**
C_15:1_ *ω6c*	**18.7**	4.5	3.4
C_15:0_	**21.6**	3.3	1.0
iso-C_16:0_	1.0	1.7	0.5
C_16:1_ *ω7c*	7.5	**16.2**	**13.5**
C_16:1_ *ω5c*	0.7	1.3	1.6
C_16:0_	4.8	5.6	3.1
iso-C_15:0_ 3-OH	2.2	7.2	**10.2**
C_15:0_ 3-OH	8.9	ND	ND
C_17:1_ *ω6c*	8.1	4.5	1.6
C_17:0_	1.9	0.5	0.3
C_16:0_ 3-OH	2.4	3.3	2.0
Summed feature 9^*^	0.6	1.0	0.3
Summed feature 11^*^	0.2	0.9	0.9

* Summed features represent groups of two or three fatty acids that could not be separated by GLC with the MIDI system: Summed features 9 consisted of iso-C_16:0_ 3-OH and/ or unknown fatty acid of ECL 17.157 DMA and Summed features 11 consisted of iso-C_17:0_ 3-OH and/ or C_18:2_ DMA.

All data listed in the table are from this study, and only those accounting for 1.0% or more in one of the strains are given. The dominant fatty acids (>10%) are in bold. ND, not detected.

## Conclusion

In this study, we revised the taxonomic positions of multiple species in the order *Geobacterales* based on whole-genome analysis. Firstly, the different genomic characteristics, particularly for genome size and G+C content, revealed that some species in the genera *Geobacter* and ‘*Pseudopelobacter*’ are distinct enough to represent novel independent genera in the family *Geobacteraceae*. Then, the multigene-based phylogenetic analyses, performed with MLSA of four housekeeping genes, UBCG of 92 core genes and GTDB of concatenated 120 ubiquitous single-copy proteins, placed the three taxonomy-pendent species (*G. argillaceus*, *G. luticola*, and *G. pelophilus*) into three monophyletic branches, which were clearly separated from their phylogenetic neighbors, indicating the higher taxonomic ranks of these three species. Moreover, these phylogenetic analyses also assigned the genus *Oryzomonas* of the family *Geobacteraceae* to the family ‘*Pseudopelobacteraceae*’, resulting in an amendment of these two families. Next, the genomic similarity indexes based on amino acid sequence and nucleic acid sequence were further calculated, which showed a high correlation with the phylogenetic structures where the relative bacterial species were clustered together. Based on the criterion that one genus should be clustered into one phylogenetic branch only, 70% of AAI, 65% of POCP, and 74% of ANI were proposed as the appropriate thresholds for genus separation within the order *Geobacterales*. Considering this, the three taxonomy-pendent species and *G. chapellei* should represent four novel genera rather than species as they do currently.

Since the genome information of the species *Geobacter psychrophilus* P35^T^ is lacking now, it was absent in the phylogenomic and genome similarity analyses. Based on the 16S rRNA gene tree, this species was closely clustered with *G. chapellei* and shared the highest similarity value (97.3%) with this species. However, as noted above, the G+C content of strain *G. psychrophilus* P35^T^ was 63.8mol%, much higher than that of strain *G. chapellei* 172^T^ (51.1%). Thus, this obvious difference in G+C content indicated that species *G. psychrophilus* does not belong to the same genus as *G. chapellei* but may represent a novel genus, including this species. However, the type strain *G. psychrophilus* P35^T^ in this genus is currently unavailable in culture collection centers, which is contrary to the rules of International Code of Nomenclature of Prokaryotes to propose a validly published genus name, thus, this species was not reclassified in this study. Taken together, the order *Geobacterales* is more diverse at the genus level than previously known and currently contains 10 genera, including four novel ones that were proposed in this study. In view of their phenotypic characteristics, we propose the reclassification of *Geobacter pelophilus* as *Geoanaerobacter pelophilus* comb. nov., *Geobacter luticola* as *Geomobilimonas luticola* comb. nov., *Geobacter argillaceus* as *Geomobilibacter argillaceus* comb. nov., and *Geobacter chapellei* as *Pelotalea chapellei* comb. nov. (Table 4).

**Table 4:** Original and proposed taxonomy of the strains in the order *Geobacterales*.

Original taxonomy			Proposed taxonomy		
Family	Genus	Species	Species	Genus	Family
*Geobacteraceae*	*Geomonas*	*G. silvestris*	–	*Geomonas*	*Geobacteraceae*
		*G. limicola*	–		
		*G. bemidjiensis*	–		
		*G. bremensis*	–		
		*G. paludis*	–		
		*G. oryzae*	–		
		*G. edaphica*	–		
		*G. ferrireducens*	–		
		*G. terrae*	–		
	*Geotalea*	*G. uraniireducens*	–	*Geotalea*	
		*G. toluenoxydans*	–		
		*G. daltonii*	–		
	*Geobacter*	*G. luticola*	*G. luticola*	***Geomobilimonas***	
		*G. argillaceus*	*G. argillaceus*	***Geomobilibacter***	
		*G. pelophilus*	*G. pelophilus*	***Geoanaerobacter***	
		*G. sulfurreducens*	–	*Geobacter*	
		*G. anodireducens*	–		
		*G. pickeringii*	–		
		*G. soli*	–		
		*G. hydrogenophilus*	–		
		*G. grbiciae*	–		
		*G. metallireducens*	–		
	*Oryzomonas*	*O. japonica*	*O. japonica*	*Oryzomonas*	*‘Pseudopelobacteraceae’*
		*O. sagensis*	*O. sagensis*		
		*O. rubra*	*O. rubra*		
*‘Pseudopelobacteraceae’*	*Trichlorobacter*	*T. lovleyi*	–	*Trichlorobacter*	
		*T. thiogenes*	–		
	*‘Pseudopelobacter’*	*Geobacter chapellei*	*P. chapellei*	***Pelotalea***	
		*Pelobacter propionicus*	–	*‘Pseudopelobacter’*	
		*Geobacter psychrophilus*	–		

–, represent unchanged strains in genus or family level reclassification. Single quotation marks indicate the invalidly published names, owing to the absence of the type strains in two different culture collection centers. The novel genera proposed in this study are in bold.

In addition, two novel strains, namely Red51^T^ and Red69^T^, were isolated from paddy soils and showed the highest 16S rRNA gene similarity to the type strains in the genus *Geomonas*. Polyphasic taxonomic analyses, including phenotypic, biochemical, and genomic characteristics, further confirmed that these two strains represent two novel species in the genus *Geomonas* of the family *Geobacteraceae*. Given their ability to fix nitrogen, we proposed their names as *Geomonas azotofigens* sp. nov. (type strain Red51^T^) and *Geomonas diazotrophica* sp. nov. (type strain Red69^T^).

Our work constructed a taxonomic framework with whole-genome-based phylogeny and similarity comparison for all currently available genome sequences of type strains within the order *Geobacterales*, which also serves as a foundation for the classification of current and future isolates within the order *Geobacterales*. Nevertheless, the threshold criteria proposed in this study for genus separation are not very strict, because several genera currently contain one type species, and the small scale of species population may lead to statistical bias in cut-off value determination. Thus, the results of this study provide a reference for the following identification of bacterial strains in the order *Geobacterales*; more bacterial species are still required for robust systematic analyses.

### Description of *Geomonas azotofigens* sp. nov.

*Geomonas azotofigens* (a.zo.to.fi’gens. N.L. neut. n. *azotum*, nitrogen; L. v. *figo*, to fix; N.L. part. adj. *azotofigens*, nitrogen-fixing).

Cells are Gram-stain negative, strictly anaerobic, non-spore-forming, rod-shaped, and motile by peritrichous flagella. Colonies are circular and red-pigmented due to the presence of *c*-type cytochromes. Growth occurs at 13–42°C (optimum, 30–33°C), at pH 5.5–8.0 (optimum, 6.5–7.0), and with 0–0.6% (w/v) NaCl (optimum, 0–0.2%). The mean generation time is 171min under optimum conditions in modified R2A broth. Cells can fix N_2_ to grow. With Fe(III)-NTA as the electron acceptor, tryptone, yeast powder, pyruvate, glucose, acetate, casamino acid, arginine, nicotinate, proline, mannitol, malate, methanol, lactate, succinate, serine, glycerol, propionate, ethanol, and isopropanol can be utilized as electron donors, but not phenol, butanol, benzaldehyde, toluene, or benzyl alcohol. With acetate as the electron donor, fumarate, Fe(III)-NTA, ferrihydrite, malate, and Fe(III) citrate can be utilized as electron acceptors, but not Fe(III) pyrophosphate, sulfur, or MnO_2_. Esterase (C4), esteraselipase (C8), acid phosphatase, leucine arylamidase, and naphthol-AS-BI-phosphohydrolase activities were present but alkaline phosphatase, trypsin, lipase (C14), valine arylamidase, α-mannosidase, cystine arylamidase, α-chymotrypsin, α-galactosidase, β-galactosidase, α-glucosidase, β-glucuronidase, β-glucosidase, N-acetyl-β-glucosaminidase, α-fucosidase, and catalase activities are absent. The major fatty acids are iso-C_15:0_, C_15:0,_ and C_15:1_ *ω6c*. The predominant quinone is MK-8.

The type strain, Red51^T^ (= MCCC 1K03693^T^=JCM 33032^T^), was isolated from paddy soil of a field in Fukuoka, Japan. The genomic DNA G+C content of type strain is 62.4%.

### Description of *Geomonas diazotrophica* sp. nov.

*Geomonas diazotrophica* (di.a.zo.tro’phi.*ca.* Gr. pref. *di*, two, double; N.L. masc. *azotum*, nitrogen; Gr. masc. adj. *trophikos*, nursing, tending or feeding; N.L. fem. adj. *diazotrophica*, one that feeds on dinitrogen).

Cells are Gram-stain negative, strictly anaerobic, non-spore-forming, rod-shaped, and motile by peritrichous flagella. Colonies are circular and red-pigmented due to the presence of *c*-type cytochromes. Growth occurs at 15–42°C (optimum, 30–33°C), at pH 5.5–8.0 (optimum, 6.5–7.0), and with 0–0.8% (w/v) NaCl (optimum, 0–0.2%). The mean generation time is 185min under optimum conditions in modified R2A broth. Cells can fix N_2_ to grow. With Fe(III)-NTA as the electron acceptor, tryptone, yeast powder, glucose, acetate, pyruvate, casamino acid, malate, nicotinate, proline, mannitol, methanol, lactate, arginine, succinate, serine, glycerol, propionate, ethanol, and isopropanol can be utilized as electron donors, but not phenol, butanol, benzaldehyde, toluene, or benzyl alcohol. With acetate as the electron donor, fumarate, Fe(III)-NTA, ferrihydrite, malate, and Fe(III) citrate can be utilized as electron acceptors, but not Fe(III) pyrophosphate, sulfur, or MnO_2_. Esterase (C4), esteraselipase (C8), acid phosphatase, leucine arylamidase, and naphthol-AS-BI-phosphohydrolase activities were present but alkaline phosphatase, trypsin, lipase (C14), valine arylamidase, cystine arylamidase, α-chymotrypsin, α-galactosidase, α-glucosidase, α-fucosidase, α-mannosidase, β-glucuronidase, β-galactosidase, β-glucosidase, N-acetyl-β-glucosaminidase, and catalase activities are absent. The major fatty acids are iso-C_15:0_ and C_16:1_ *ω7c*. The predominant quinone is MK-8.

The type strain, Red69^T^ (= MCCC 1K04207^T^=NBRC 114065^T^), was isolated from paddy soil of a field in Saga, Japan. The genomic DNA G+C content of type strain is 61.9%.

### Description of *Geoanaerobacter* gen. nov.

*Geoanaerobacter* [Ge.o.an.ae.ro.bac’ter. Gr. fem. n. *ge*, the earth; Gr. pref. *an*-, not; Gr. masc. n. *aer* (gen. *aeros*), air; N.L. masc. n. *bacter*, rod, staff; N.L. masc. n. *Geoanaerobacter*, an anaerobic rod from the earth.]

The description of the genus is based on the description of *Geoanaerobacter pelophilus* (Straub and Buchholz-Cleven, 2001). Gram-stain negative, strictly anaerobic, slightly curved rods, non-spore-forming, non-motile and tend to form aggregates. Multiplication by binary fission. Colonies are red-pigmented due to the presence of *c*-type cytochromes. Electron donors utilized are hydrogen, fumarate, succinate, formate, pyruvate, propionate, malate, acetate, ethanol and propanol. Electron acceptors utilized are Fe(III), Mn(IV), S^0^, malate, and fumarate. The genomic G+C content is 53.1%.

Type species: *Geoanaerobacter pelophilus*.

### Description of *Geoanaerobacter pelophilus* comb. nov.

*Geoanaerobacter pelophilus* (pe.lo’phi.la. Gr. masc. n. *pelos* mud; Gr. masc. adj. *philos* loving; N.L. masc. adj. *pelophilus*, mud-loving, as this species was isolated from freshwater mud.)

Basonym: *Geobacter pelophilus* (Straub and Buchholz-Cleven, 2001).

The description is as given by Straub and Buchholz-Cleven (2001) with the following modification. The genomic G+C content is 53.1%. The genome size is 4.4 Mbp. The accession number for the whole genome sequence of strain DSM 12255^T^ is JAHCVJ000000000. The type strain Dfr2^T^ (= ATCC BAA-603^T^=DSM 12255^T^) was isolated from a freshwater ditch in Bremen, Germany.

### Description of *Geomobilimonas* gen. nov.

*Geomobilimonas* (Ge.o.mo.bi.li.mo’nas. Gr. fem. n. *ge*, the earth; L. masc. adj. *mobilis*, mobile; L. fem. n. *monas*, a unit, monad; N.L. fem. n. *Geomobilimonas*, a mobile monad from the earth.)

The description of the genus is based on the description of *Geomobilimonas luticola* (Viulu et al., 2013). Gram-stain negative, straight singular rods, and motile with a flagellum. With Fe(III)-NTA as electron acceptor, acetate, lactate, pyruvate, and succinate are utilized as electron donors, but not H_2_, formate, benzoate, fumarate, propionate, methanol, butyrate, toluene, butanol, ethanol, benzoate, glucose, phenol, or methane. With acetate as an electron donor, amorphous iron (III) hydroxide, ferric citrate, and nitrate are reduced as electron acceptors, but not sulfate, fumarate, or malate. Major respiratory quinone is MK-8. The genomic G+C content is 58.2%.

Type species: *Geomobilimonas luticola*.

### Description of *Geomobilimonas luticola* comb. nov.

*Geomobilimonas luticola* [lu.ti’co.la. L. n. *lutum* mud; L. suff. -*cola* (from L. n. *incola*) inhabitant, dweller; N. L. n. *luticola* the mud dweller, the type strain of this species was isolated from mud of lotus field.]

Basonym: *Geobacter luticola* (Viulu et al., 2013).

The description is as given by Viulu et al. (2013) with the following modification. The genomic G+C content is 58.2%. The genome size is 3.7 Mbp. The accession number for the whole genome sequence of strain JCM 17780^T^ is JAHCVK000000000. The type strain OSK6^T^ (= DSM 24905^T^=JCM 17780^T^) was isolated from a lotus field in Aichi prefecture, Japan.

### Description of *Geomobilibacter* gen. nov.

*Geomobilibacter* (Ge.o.mo.bi.li.bac’ter. Gr. fem. n. *ge*, the earth; L. masc. adj. *mobilis*, mobile; N.L. masc. n. *bacter*, rod, staff; N.L. masc. n. *Geomobilibacter*, a mobile rod from the earth.)

The description of the genus is based on the description of *Geomobilibacter argillaceus* (Shelobolina et al., 2007). Cells are Gram-negative, motile, regular rods. Cells have one lateral flagellum. Uses PCFO, Fe(III) NCA, Fe(III) pyrophosphate, ferric citrate, MnOOH, nitrate and elemental sulfur as electron acceptors. Reduces U(VI) in cell suspension. Oxidizes the following electron donors: acetate, ethanol, lactate, butanol, glycerol, pyruvate, butyrate, and valerate. The genomic G+C content is 58.2%.

Type species: *Geomobilibacter argillaceus*.

### Description of *Geomobilibacter argillaceus* comb. nov.

*Geomobilibacter argillaceus* (ar.gil.la’ce.us. L. masc. adj. *argillaceus* of clay).

Basonym: *Geobacter argillaceus* (Shelobolina et al., 2007).

The description is as given by Shelobolina et al. (2007) with the following modification. The genomic G+C content is 58.2%. The genome size is 4.4 Mbp. The accession number for the whole genome sequence of strain G12^T^ is VLLN00000000.1. The type strain, G12^T^ (= ATCC BAA-1139^T^=JCM 12999^T^), was isolated from subsurface kaolin strata in Georgia, United States.

### Emended Description of the Family Geobacteraceae

The description of the family remains as given by Holmes et al. (2004a) with the following emendations. The family currently contains six genera *Geomonas*, *Geotalea*, *Geobacter*, *Geoanaerobacter*, *Geomobilimonas,* and *Geomobilibacter*, which consist of an independent branch in the order *Geobacterales* based on the phylogenomic trees.

### Description of *Pelotalea* gen. nov.

*Pelotalea* (Pe.lo.ta’le.a. Gr. masc. n. *pelos*, mud; L. fem. n. *talea*, a rod; N.L. fem. n. *Pelotalea*, a mud inhabiting rod.)

The description of the genus is based on the description of *Pelotalea chapellei* (Coates et al., 2001). Cells are Gram-stain negative, rod-shaped, non-spore-forming, and non-motile. Strictly anaerobic chemo-organotroph that oxidizes acetate with the concomitant reduction of Fe(III). Other electron donors used in addition to acetate include lactate, ethanol, and formate. Electron acceptors used include Mn(IV), Fe(III), fumarate, and the humic-substance analogue 2,6-anthraquinone disulfonate; it does not use Fe(III) chelated with citrate. The cells contain *c*-type cytochromes. The genomic G+C content is 51.1%.

Type species: *Pelotalea chapellei*.

### Description of *Pelotalea chapellei* comb. nov.

*Pelotalea chapellei* (cha.pel’le.i. N.L. gen. masc. n. *chapellei* of Chapelle, named after Frank Chapelle, who contributed to our knowledge of subsurface biogeochemistry).

Basonym: *Geobacter chapellei* (Coates et al., 2001).

The description is as given by Coates et al. (2001) with the following modification. The genomic G+C content is 51.1%. The genome size is 3.9 Mbp. The accession number for the whole genome sequence of strain DSM 13688^T^ is JAHDYS000000000. The type strain, 172^T^ (= ATCC 51744^T^=DSM 13688^T^), was isolated from Fe(III)-reducing enrichments of subsamples from deep aquifer sediments of the Atlantic Coastal Plain in South Carolina, United States.

### Emended Description of *Geobacter hydrogenophilus*

The description is as given by Coates et al. (2001) with the following modification. The genomic G+C content is 59.6%. The genome size is 4.0 Mbp.

The accession number for the whole genome sequences of the type strain DSM 13691^T^ is JAHCZI000000000.

### Emended Description of *Geobacter grbiciae*

The description is as given by Coates et al. (2001) with the following modification. The genomic G+C content is 59.5%. The genome size is 4.2 Mbp.

The accession number for the whole genome sequences of the type strain DSM 13689^T^ is JAHDIW000000000.

## Data Availability Statement

The GenBank accession numbers for the 16S rRNA gene sequences of strains Red51^T^ and Red69^T^ are MH915554 and MH915555, respectively. The Whole Genome Shotgun projects of Geobacterales type strains have been deposited at DDBJ/ENA/GenBank under the accession numbers as follows: *Geom. azotofixans* Red51^T^ (JAHLME000000000), *Geom. diazotrophicus* Red69^T^ (JAHLMF000000000), *Geob. pelophilus* DSM 12255^T^ (JAHCVJ000000000), *Geob. luticola* JCM 17780^T^ (JAHCVK000000000), *Geob. hydrogenophilus* DSM 13691^T^ (JAHCZI000000000), *Geob. grbicium* DSM 13689^T^ (JAHDIW000000000), and *Geob. chapellei* DSM 13688^T^ (JAHDYS000000000).

## Author Contributions

ZX, YM, KS, and HI designed the experiment and edited the manuscript. ZX performed most of the laboratory work and data analysis, and wrote the manuscript. HI and YS collected the soil samples. HI performed the isolation work. XW detected the bacterial biomass. NU took the TEM images. All authors contributed to the article and approved the submitted version.

## Funding

This study was supported by JSPS KAKENHI Grant Numbers JP20H00409, JP20H05679, JP20K15423, JP18K19165, JP18K14366, and JP17H01464, The Canon Foundation, and JST-Mirai Program Grant Number JPMJMI20E5. ZX also thanks financial support by the China Scholarship Council (CSC).

## Conflict of Interest

The authors declare that the research was conducted in the absence of any commercial or financial relationships that could be construed as a potential conflict of interest.

## Publisher’s Note

All claims expressed in this article are solely those of the authors and do not necessarily represent those of their affiliated organizations, or those of the publisher, the editors and the reviewers. Any product that may be evaluated in this article, or claim that may be made by its manufacturer, is not guaranteed or endorsed by the publisher.

## Footnotes

^1^ https://lpsn.dsmz.de/genus/geomonas

^2^ http://enve-omics.ce.gatech.edu/

^3^ https://github.com/2015qyliang/POCP

^4^ http://textbookofbacteriology.net/growth_3.html
