# Supplementary material for: Genome-Based Taxonomic Rearrangement of the Order Geobacterales Including the Description of Geomonas azotofigens sp. nov. and Geomonas diazotrophica sp. nov
Source: Front Microbiol. 2021 Sep 30;12:737531. doi: 10.3389/fmicb.2021.737531 (PMC8516083; doi:10.3389/fmicb.2021.737531)
Supplement: Supplementary file 1 [file Data_Sheet_1.docx]

**
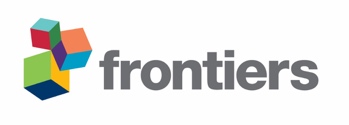
**

***Supplementary Material***

**Genome-based taxonomic ﻿rearrangement of the order** ***Geobacterales* including the description of** ***Geomonas*** ***azotofigens* sp. nov. and *Geomonas diazotrophica* sp. nov.**

Zhenxing Xu^1*^, Yoko Masuda^1^^*^, ﻿Xueding Wang^1^, Natsumi Ushijima^2^, Yutaka Shiratori^3^, ﻿Keishi Senoo^1,4^,﻿ Hideomi Itoh^5^

﻿^1^Department of Applied Biological Chemistry, Graduate School of Agricultural and Life Sciences, The University of Tokyo, Tokyo, Japan

﻿^2^Support Section for Education and Research, Graduate School of Dental Medicine, Hokkaido University, Hokkaido, Japan

^3^Niigata Agricultural Research Institute, Niigata, Japan

^4^Collaborative Research Institute for Innovative Microbiology, The University of Tokyo, Tokyo, Japan

^5^Bioproduction Research Institute, National Institute of Advanced Industrial Science and Technology (AIST) Hokkaido, Hokkaido, Japan

Author for correspondence:

Zhenxing Xu, Ph.D.

Email: xuzx.ut@gmail.com

Yoko Masuda, Ph.D.

Email: [ygigico@gmail.com](mailto:ygigico@gmail.com)

**Supplementary table:**

Supplementary Table 1. Annotated genes involved in nitrogen fixation and assimilatory sulfate reduction of the two isolated strains.

| Gene symbol | Annotation | Accession numbers in NCBI database | |
| --- | --- | --- | --- |
|  |  | Red51^T^ | Red69^T^ |
| **Nitrogen fixation gene** | | | |
| *nifS* | Cysteine desulfurase | MBU5613333.1 | MBU5636115.1 |
| *nifU* | Iron-sulfur cluster assembly scaffold protein | MBU5612781.1  MBU5613332.1 | MBU5636116.1  MBU5636939.1 |
| *nifB* | Nitrogenase FeMo-cofactor synthesis FeS core scaffold and assembly protein | MBU5613235.1 | MBU5637568.1 |
| *nifX* | Nitrogenase FeMo-cofactor carrier protein | MBU5614029.1 | MBU5637573.1 |
| *nifB-2* | NifB-domain protein, type 2 | MBU5614027.1 | MBU5637571.1 |
| *nifEN* | Nitrogenase FeMo-cofactor scaffold and assembly protein | MBU5614030.1 | MBU5637574.1 |
| *nifV* | Homocitrate synthase | MBU5614034.1 | MBU5637578.1 |
| *nifH* | Nitrogenase (molybdenum-iron) reductase and maturation protein | MBU5613234.1  MBU5614033.1 | MBU5637577.1 |
| *nifD* | Nitrogenase (molybdenum-iron) alpha chain | MBU5614032.1 | MBU5637576.1 |
| *nifK* | Nitrogenase (molybdenum-iron) beta chain | MBU5614031.1 | MBU5637575.1 |
| *vnfE* | Nitrogenase vanadium-cofactor synthesis protein | MBU5613248.1 | - |
| *vnfN* | Nitrogenase vanadium-cofactor synthesis protein | MBU5613249.1 | - |
| **Assimilatory sulfate reduction** | | | |
| *cysNC* | Bifunctional enzyme CysN/CysC | MBU5613655.1 | MBU5637257.1 |
| *cysD* | Sulfate adenylyltransferase | MBU5613654.1 | MBU5637258.1 |
| *cysH* | Phosphoadenosine phosphosulfate reductase | MBU5613653.1 | MBU5637259.1 |
| *sir* | Sulfite reductase (ferredoxin) | MBU5612111.1 | MBU5635242.1 |

**Supplementary figures:**

**Supplementary Figure 1.** The growth curves of the two isolated strains Red51^T^ and Red69^T^. Cells were grown on R2A broth supplemented with 5 mM fumarate at 30 °C with 1/100 inoculation scale. The range of exponential phase is from 16^th^ hour to 24^th^ hour. Data were all presented as means ± standard deviations (SD) of triplicate. When not shown, error bars are smaller than the symbol size.

**Supplementary Figure 2.** ﻿ Heatmap of 16S rRNA gene similarity values for all type strains in the order *Geobacterales*.

**Supplementary Figure 3.** ﻿ Heatmap of average amino acid identity (AAI) values in upper-right and average nucleotide identity (ANI) values in lower-left for 31 genomes of bacteria in the order *Geobacterales*.

**Supplementary Figure 4.** ﻿ Heatmap of the percentage of conserved protein (POCP) values for 31 genomes of bacteria in the order *Geobacterales*.


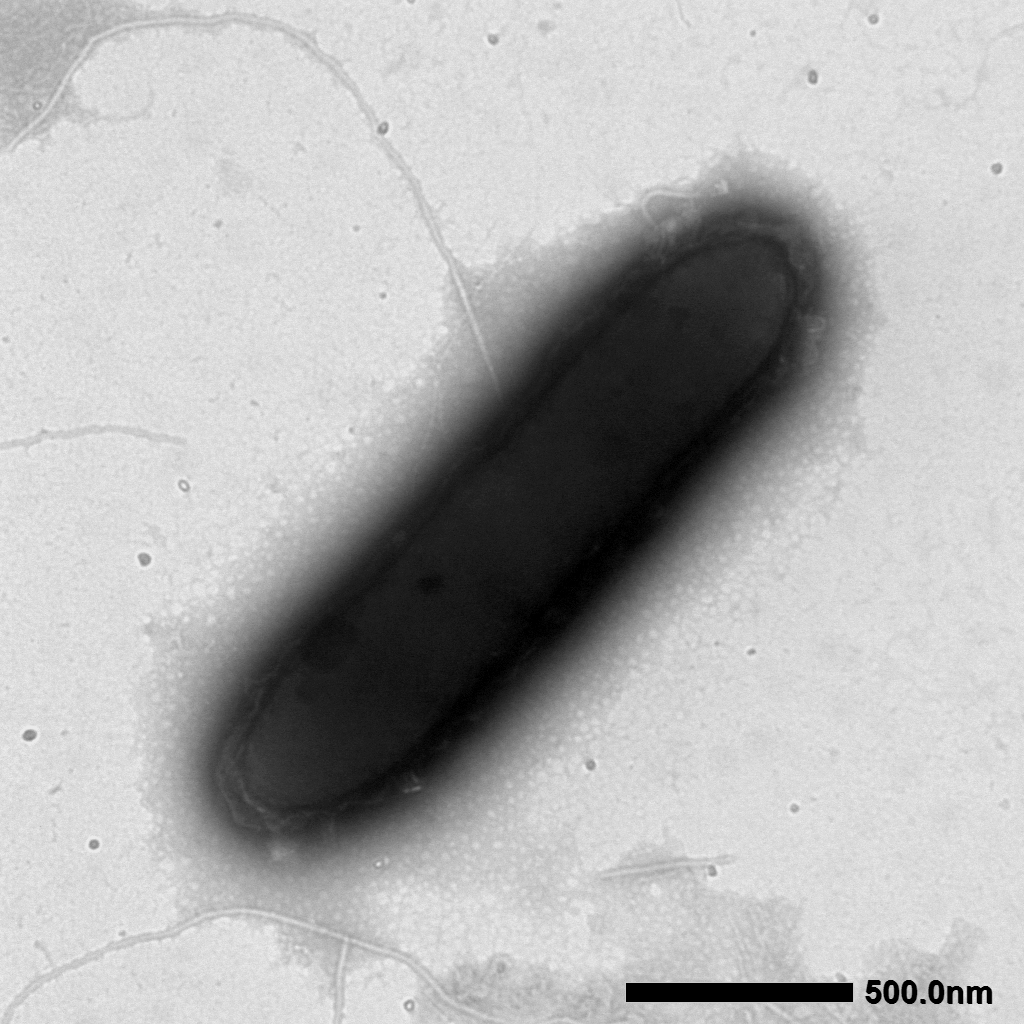

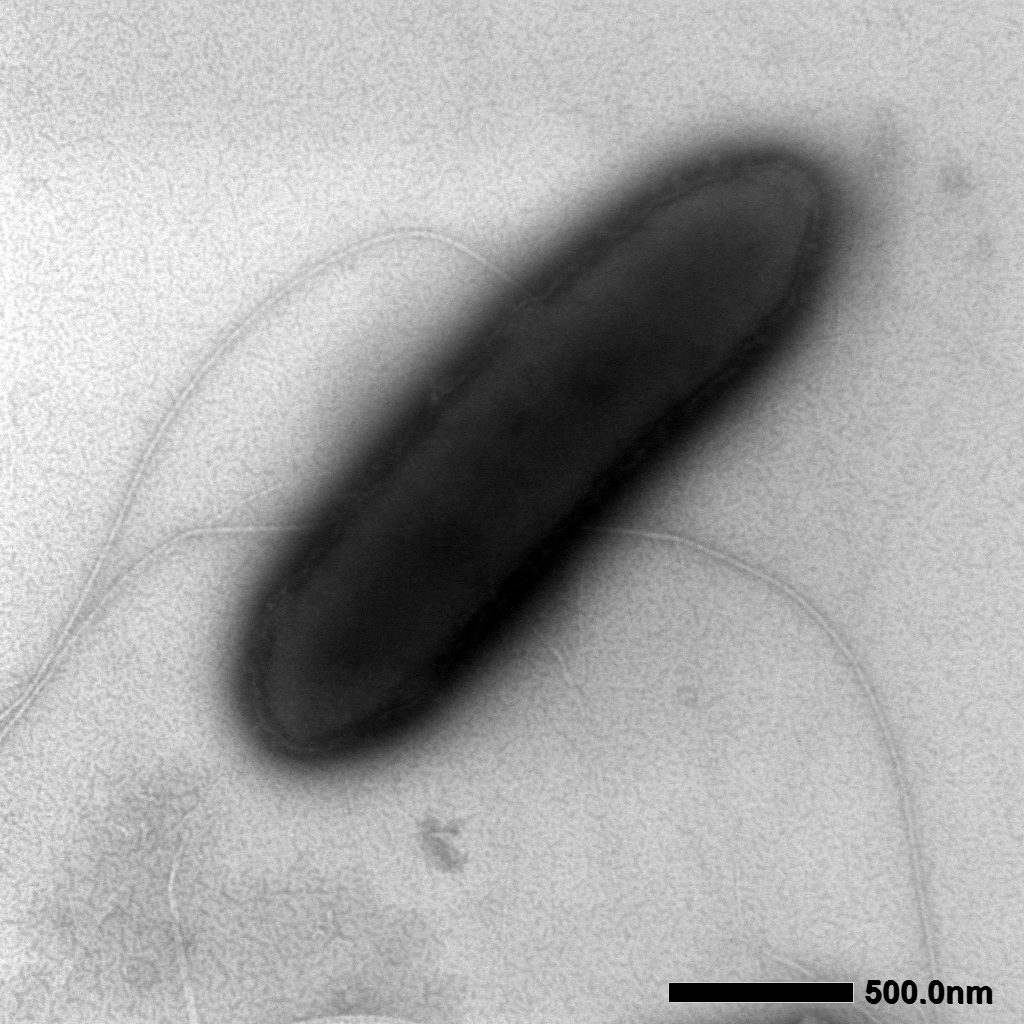


**B**

**A**

**Supplementary Figure 5.** ﻿Transmission electron micrograph (TEM) images of the two isolated strains. A, strain Red51^T^; B, strain Red69^T^. Bar. 500 nm.

**Supplementary Figure 6.** The growth curves of the two isolated strains with N_2_ or NH_4_^+^ as the nitrogen source. A, strain Red51^T^; B, strain Red69^T^. ﻿Data were all presented as means ± standard deviations (SD) of triplicate.

Peaks: 424, 524, 554 nm

Peaks: 426, 524, 554 nm

**B**

**A**

**Supplementary Figure 7.** Difference spectrum of whole cells for the two isolated strains in the wavelength range of 400-800 nm. A, strain Red51^T^; B, strain Red69^T^. The values shown in the figures were wavelength of every peak.
